# Assessing sound symbolism: Investigating phonetic forms, visual shapes and letter fonts in an implicit bouba-kiki experimental paradigm

**DOI:** 10.1371/journal.pone.0208874

**Published:** 2018-12-21

**Authors:** Léa De Carolis, Egidio Marsico, Vincent Arnaud, Christophe Coupé

**Affiliations:** 1 Laboratoire Dynamique du Langage, CNRS & Université de Lyon, Lyon, France; 2 Département des arts et lettres, Université du Québec à Chicoutimi, Chicoutimi, Canada; 3 Department of Linguistics, The University of Hong Kong, Hong Kong SAR, China; University of Texas at El Paso, UNITED STATES

## Abstract

Classically, in the bouba-kiki association task, a subject is asked to find the best association between one of two shapes–a round one and a spiky one–and one of two pseudowords–bouba and kiki. Numerous studies report that spiky shapes are associated with kiki, and round shapes with bouba. This task is likely the most prevalent in the study of non-conventional relationships between linguistic forms and meanings, also known as sound symbolism. However, associative tasks are explicit in the sense that they highlight phonetic and visual contrasts and require subjects to establish a crossmodal link between stimuli of different natures. Additionally, recent studies have raised the question whether visual resemblances between the target shapes and the letters explain the pattern of association, at least in literate subjects. In this paper, we report a more implicit testing paradigm of the bouba-kiki effect with the use of a lexical decision task with character strings presented in round or spiky frames. Pseudowords and words are, furthermore, displayed with either an angular or a curvy font to investigate possible graphemic bias. Innovative analyses of response times are performed with GAMLSS models, which offer a large range of possible distributions of error terms, and a generalized Gama distribution is found to be the most appropriate. No sound symbolic effect appears to be significant, but an interaction effect is in particular observed between spiky shapes and angular letters leading to faster response times. We discuss these results with respect to the visual saliency of angular shapes, priming, brain activation, synaesthesia and ideasthesia.

## Introduction

Sound symbolism refers to the broad hypothesis that some phonetic units intrinsically carry semantic content. One of the best-known experimental evidences in favor of sound symbolism emergence is the so-called bouba-kiki effect. It consists in the presentation of two shapes, a curvy and a spiky one, and of two pseudowords, ‘bouba’ and ‘kiki’ (or ‘maluma’ and ‘takete’ in Köhler [1]’s original experiment). The subject has to select their preferred association between a shape and a pseudoword during a forced-choice task. Many studies show the same pattern of response: ‘bouba’ is more often associated with round shapes, while ‘kiki’ is more often associated with spiky shapes. This has been demonstrated with people from different countries and speaking different languages, using different kinds of phonetic and visual materials [2–6]. The effect is also discussed in infants [7]. Rogers and Ross reported no preferential association in the Songe people of New-Guinea [8]. Their study, however, lacks a precise description of the protocol and information such as the number of persons surveyed. Overall, the results suggest that these sound symbolic associations are a near-universal trend in human populations.

In 2005, Westbury [9] shifted from the classical explicit association task to a more implicit paradigm assessing sound symbolism: a lexical decision task where written forms were presented in either spiky or rounded frames. The general purpose of the present study is to extend this experiment. In the section below, four core components of the approach are discussed in the light of recent studies: (1) which phonemes and features get associated with visual shapes; (2) the role played by contrasts in association tasks; (3) the transparency of the tasks used in the field of sound symbolism, and its influence on the strength of associations; (4) the possible influence of the graphemic shape of letters when written forms are part of the experimental setting.

## Rationale

### Sound symbolism of consonants and vowels

A number of psycholinguistic studies have refined the phonetic properties involved in sound symbolism. An early question has been the relative weight of consonants and vowels in subjects’ preferred associations. In the 1970s, Tarte [5] argued for a greater influence of vowels while testing a small number of pseudowords and phonemes. A precise assessment of the implication of vocalic features was, furthermore, provided by Knoeferle, Li, Maggioni & Spence [10]. Departing from Tarte’s statement, however, recent studies have overall ascertained the predominant role of consonants [6,11,12]. Although with few subjects, Ahlner and Zlatev [13] have also argued that vowels and consonants have distinct but complementary roles. More precisely, in their study, the vowel and the consonant of a CVCV pseudoword could be either congruent or incongruent with respect to what they associated with at the sound symbolic level. In the congruent case, no statistically significant difference was observed between associations involving pseudowords differing on both consonant and vowel (e.g. /titi/ vs /lulu/) and associations involving pseudowords differing on either of them (e.g. /kiki/ vs /nini/ and /lili/ vs /lulu/). In the incongruent case (eg., /tutu/ vs /lili/), the associations were primarily explained by the consonant, although the associative bias did not reach statistical significance.

It is difficult to know precisely what features of consonants trigger associations. Generally speaking, plosives have been shown to associate with spiky shapes, and sonorants with rounded shapes. There are, however, methodological issues, namely the choice of various subsets of consonants in the broad categories of plosives and continuants, and their potentially unbalanced contrasts in the construction of experimental pseudowords. Both can mask which phonetic contrasts subjects rely on in their answers. It is thus hard to disentangle the role of each consonantal feature, among others manner, place of articulation and voicing–the three main features of consonants. For example, Nielsen and Rendall [6,14] contrasted voiceless plosives ([p, t, k]) with sonorants ([l, m, n]), making it hard to judge whether voicing alone, manner alone or both of them have significant effects. As suggested more recently by Nobile [15], manner of articulation and voicing may in fact independently influence subjects’ patterns of answers (see Table 1 below). This author tested phonetic contrasts along a number of articulatory dimensions with a 2×2 association task (two visual stimuli and two pseudowords). Confounding phonetic dimensions were, however, present in some of the contrasts, for example voicing and place of articulation in the contrast between plosives and fricatives.

**Table 1:** Nobile [15]’s results of sound symbolic associations between consonant features and visual shapes.

		Visual shape	
		Spiky	Round
**Phonetic features**	**Voicing**	Voiceless obstruents (plosives & fricatives)	Voiced obstruents (plosives & fricatives)
	**Manner**	Plosives (voiced & voiceless)	Fricatives (voiced & voiceless)
	**Manner**	Oral (fricatives)	Nasal (sonorants)
	**Place**	Palato-velar (plosives & fricatives)	Alveo-dental (plosives & fricatives)

### Contrasts in association tasks

With respect to the former question of the phonetic features involved in sound symbolism, the contrastive or non-contrastive nature of the proposed task may have an influence on the associations favored by subjects. If a contrast between two sound forms is presented to the subject in a 1×2 (one visual stimulus and two pseudowords) or in a 2×2 association task, a comparison may take place *between* contrasted sounds or phonetic features in order to choose the more appropriate *with respect to the other*, *much along the lines distinctive features differentiate between phonemes*. As an example, let’s assume that when presented with /d/ and /t/ in a 1×2 bouba-kiki association task, subjects associate preferentially /d/ with round shapes, and /t/ with spiky ones. Let’s also assume that when presented with /d/ and /n/, they associate preferentially /n/ with round shapes, and /d/ with spiky shapes. Comparing both results, what is associated to /d/ depends on the contrast created between it and another consonant. A logical deduction would then be that /t/, /d/ and /n/ can be placed along a continuum, with /t/ and /n/ at the extremities and /d/ between them.

What if /t/, /d/ and /n/ are now presented independently, i.e. without contrast, in a 2×1 (two visual stimuli and one pseudoword) association task? It may be here risky to straightforwardly anticipate the results from the previous ones: any of these three segments may turn to associate preferentially with either round shapes or spiky shapes, or show no significant pattern of association. Indeed, what is tested here is now phonetically intrinsic relationships between sound forms and shapes, not relative ones. One could, however, suggest from the previous results that /t/ will be more associated with spiky shapes, and /n/ with round shapes—/d/ is more elusive.

Relating to Nobile’s results on the independent effects of voicing and manner, it is unsurprising that voiced plosives like /d/ are harder to assess. On the one hand, plosives are associated with spiky shapes while sonorants are associated with round ones; on the other hand, voiced consonants are associated with round and voiceless ones with spiky. If a contrastive presentation of two pseudowords sheds light on one of these two characteristics, it can be predicted that /d/ will be more associated with round shapes when presented along with a voiceless plosive such as /t/, and more associated with spiky shapes when presented with a voiced sonorant such as /n/. But it remains difficult to predict what will happen when /d/ is presented alone in 1×1 or 2×1 association tasks, since this will likely depend on the relative associative strengths of the competing features–voicing and manner (letting aside further possible interactions with vowels). Additionally, the fine graphic details of the used figures will also play a significant role. All in all, the precise nature of the task must be factored in in the analysis of the results.

### Explicit versus implicit tasks to assess sound symbolism

Nielsen and Rendall [14] have argued that the strength of the bouba-kiki effect is overestimated because of the ‘transparency’ of the testing protocols. Indeed, associative tasks point to sound symbolism when requesting the subjects to establish a link between stimuli of different natures. As previously said, they also point to phonetic and/or visual contrasts when asking explicitly to choose between two stimuli of the same nature. Transparent presentations of contrasts may lead to metacognitive strategies masking more low-level processes and increasing effect sizes.

The previous consideration suggests why Nielsen and Rendall got smaller effect sizes for sound symbolic associations brought to light in their implicit experimental protocol. It consisted in learning pairs of shapes (rounded or spiky) and pseudowords (composed of either voiceless plosives [p, t, k], or sonorants [l, m, n]). In the first part of the experiment, half of the participants learnt ‘congruent’ pairs (with an assessment of congruence coming from earlier studies), the other half ‘incongruent’ pairs. Then, in the second part of the experiment, other pairs were presented and subjects had to decide whether these pairs were correct according to the rules they had previously learned. The recall performance was better in the congruent condition (53.3% vs 50.4%), which suggests that the congruent pairs were easier to learn and to remember.

A number of further studies have aimed at assessing sound symbolism in a more implicit way than ‘classical’ judgment or association tasks.

In a first study, Aveyard [11] asked participants to decide which of two images best associated with a pseudoword presented orally. A feedback was provided after each response, stating whether the association was correct or incorrect. Stimuli were presented repeatedly and the associations to be learnt were consistent throughout the experiment, but half of them were congruent at the sound symbolic level, and the other half was not. Participants could therefore not generalize sound symbolic rules for the whole set of associations. Given this, a relatively better learning performance was observed when rules were congruent (57% vs 50%).

In a subsequent study, which also consisted in a choice between two shapes for a pseudoword presented orally, subjects had to implicitly detect which shape was consistently associated with a given pseudoword [16]. This shape, e.g. a round shape, was either associated with a second shape of opposite nature, e.g. a spiky shape, or a distractor, e.g. a different round shape. Neither explicit rules nor feedbacks were given. Four learning blocks followed one another, and a quicker improvement for congruent associations was observed (from 55% vs 52% for congruent and incongruent associations in the first block to 68% vs 58% in the second block, as extracted from the figures of the article), although performance was similar at the end for congruent and incongruent pairings (70% vs 71% in the last block).

In an another study, Sidhu and Pexman [17] demonstrated the impact of the supraliminal priming of a pseudoword on the categorization of ambiguous shapes. In the written condition of the task, shapes were more categorized as round when preceded by a pseudoword composed of ‘round’ phonemes including consonants /b, m/ rather than of ‘sharp’ phonemes including consonants /t, k/ (57% vs 50%) (p. 1971–1973). This result was replicated in the oral condition (53% vs 43%).

In these three recent experiments, the effect sizes suggest altogether that the sound symbolic associations were much weaker compared to what is commonly observed in the classical explicit association tasks. However, it can be argued that the existence of metacognitive strategies cannot be ruled out. In Sidhu and Pexman’s study in particular, pseudowords were consciously perceived and the justification given for their presence–they were described as not relevant to the current task but to later ones–could easily be questioned by participants.

As already mentioned, Westbury [9] conducted a study with English speakers using a protocol that can be considered as significantly more implicit than the previous ones. A lexical decision task was conducted with written words and pseudowords composed of either or both plosives or sonorants (Westbury actually uses the terms stops and continuants, following a phonological distinction rather than the phonetic distinction we adopt in this paper; both descriptions are valid, as explained in [18]), presented in a spiky or a rounded frame. Response time for pseudowords composed of plosives were significantly faster when presented in spiky frames, and conversely, responses of pseudowords composed of sonorants were significantly faster when presented in rounded frames. In a second task, letters and numbers were tested in the same manner. Decisions on these items did not require the same semantic access, and hence allowed to evaluate lower-level cognitive processes. In both experiments, results were consistent with sound symbolic expectations, i.e. an interaction was observed between the shape of frames and the type of consonants. However, effects were only marginally significant, suggesting once again that the less transparent a protocol, the weaker the sound symbolic associations.

### Influence of the shape of letters in tasks on sound symbolism

In all studies focusing on the sound symbolism of graphic shapes but relying on written forms, a potential confound exists given possible intra-modal visual associations involving the graphemic shapes of the written forms. This issue has been noted in some of the aforementioned studies. Westbury [9] assessed the influence of the shape of letters and numbers in his second task, distinguishing angular characters from curvy ones. He noticed no interaction between graphemic features and frames, which led him to conclude that there was a ‘*lack of evidence to support the orthographic form hypothesis*’ (p. 16), although it can be argued that the second task was in many ways different from the first lexical decision task. As for Nielsen and Rendall [14], they neutralized the issue by creating ‘mixed orthographic representations’, with lowercase and uppercase letters, that ‘did not consistently align with either possible matching rule’ (p. 119).

The fact that a lot of studies actually presented the linguistic material orally [5,6,11–13,16,17] is obviously a strong point in favor of sound symbolism. One could argue that acoustic stimuli activate written representations in the subjects’ minds, at least in the minds of the competent readers, usually university students, that form the bulk of participants in experimental psycholinguistics. This cannot however be the whole story, given Bremner et al. [2]’s results with a “bouba-kiki” task in a population without written tradition, the Himbas of Namibia. While it makes perfect sense to prevent a writing bias when investigating sound symbolism, doing so however restricts our understanding of possibly intertwined processes, implying both sound symbolic associations and purely visual ones.

Cuskley, Simner and Kirby [19] attempted to explain the bouba-kiki phenomenon in terms of graphemic bias rather than, or in addition to, sound symbolism. With written pseudowords, they found that angular letters (*k*, *t*, *z* or *v*) associate with spiky shapes, while curvy letters (*g*, *d*, *s*, *f*) associate with rounded shapes. Interestingly, this effect persisted with oral pseudowords, which could possibly suggest that hearing a pseudoword automatically activate the mental representation of its written form, although other explanations are possible, as mentioned in the next two paragraphs. The authors additionally found an interaction between voicing and shape (voiced consonants with round shapes, and voiceless consonants with spiky shapes), in the oral condition only. These results do not challenge preceding results, but highlight that it is indeed not easy to separate sound symbolic associations from purely visual ones when using written material.

Along the same line, Westbury [9] noted that disentangling concurrent effects–purely graphic and sound symbolic—is difficult. This starts with the difficulty of judging whether a graphemic symbol is more angular or curvy–for example, “f” is considered as angular by Westbury and as curvy by Cuskley, et al. [19]). Additionally, associations between i) curvy letters and round shapes, and ii) angular letters and spiky shapes may also reflect intricate phonetic properties of the corresponding sounds. /d/, /g/, /s/ and /f/ may be related to round shapes because voiced plosives and voiceless fricatives are. Conversely, /t/, /k/, /z/ and /v/ may be related to spiky shapes because voiceless plosives and voiced fricatives are. This is actually supported by Fort et al. [12]’s results in a purely auditory task.

Finally, phonetic features may partly decide of the graphemic forms of letters, as discussed by Turoman and Styles [20]. These authors obtained better-than-chance performance in a task that consisted in guessing which glyph among two was referring to the sound /i/ or /u/ in multiple written traditions. This suggests that the shapes of letters may be historically motivated by the sound they refer to, which would then be another instance of sound symbolism.

If they exist, intra-modal visual interactions may appear in addition to sound symbolic effects. The question is then raised of the respective effect sizes of these effects. The near-absence of significant sound symbolic effects in Cuskley et al.’s statistical models could be explained by the intricacies of an unbalanced experimental material and specific choices of consonants, e.g. choosing only fricatives [s, z, f, v] for continuants, while other studies mostly consider sonorants like [l, m, n].

Because of such difficulties, a more encompassing test of various associative effects is needed, which explicitly allows for effects that add to each other, or compete with each other.

## Method

### Ethics statement

This research has been approved by the ethical committee “Comité de Protection des Personnes SUD-EST IV” (Lyon, France) with the reference number L15-210. All participants gave written informed consent to participate in the experiment.

### Overview

Our objectives were i) to assess sound symbolism in a non-transparent task to address the issue of possible metacognitive strategies and oversized effects, ii) to pay specific attention to the involved phonetic dimensions in order to better assess their respective roles, and iii) to explicitly tackle the possible effects of written forms. We thus chose to extend rather than to replicate Westbury [9]’s experiment by adding a third independent variable to his original design: the shape of letters, using either a curvy font, *Gabriola*, or an angular font, *Agency FB*, for the display of words and pseudowords.

Furthermore, we applied some modifications to Westbury’s experimental setting. First, aiming to better disentangle the phonetic dimensions at play, we dissociated voiced and voiceless plosives, on the basis of Nobile [15]’s findings (see Table 1). Second, although they have been used in Westbury’s study and in a few others [21], we did not create mixed pseudowords (i.e. composed of both plosives and sonorants) because of our lack of expectation in this case with respect to the two unmixed conditions.

We therefore investigated the effects of three parameters with a 3×2×2 plan: the category of consonants (voiced plosives, voiceless plosives and sonorants), the type of frames (spiky or round) and the font (angular or curvy). In our analyses, we allowed for the possibility of additive effects, either superimposing or competing, and we considered pseudowords and words independently, having in mind the well-known result that expert readers do not process the former the same way as the latter [22].

Throughout the paper, the *p*-values report the ‘exact level of significance, calculated from the data after the experiment’ [23] and no arbitrary level (such as 0.05) in hypothesis testing is indicated.

### Hypotheses

Based on Westbury [9]’s experiment, we could expect an interaction between the shapes of frames and the category of consonants. More precisely, faster response times were expected in congruent situations than in incongruent situations, as specified in part (a) of Table 2. Based on Nobile’s findings, it was more difficult to formulate predictions in the case of voiced plosives, as they could be associated both with spiky frames as plosives and with round shapes as voiced consonants.

**Table 2:** Congruent and incongruent associations of visual and phonetic stimuli according to previous studies.

	Interaction of parameters under study	Type of association of stimuli	
**(a)**	**Type of frames × Category of consonants** Sound symbolic interaction	**Congruent**	spiky frames & voiceless plosives
			round frames & sonorants
		**Incongruent**	round frames & voiceless plosives
			spiky frames & sonorants
**(b)**	**Type of frames × Font** Visuo-visual interaction	**Congruent**	spiky frames & angular font
			round frames & curvy font
		**Incongruent**	round frames & angular font
			spiky frames & curvy font
**(c)**	**Category of consonants × Font** Sound symbolic interaction	**Congruent**	angular font & voiceless plosives
			curvy font & sonorants
		**Incongruent**	curvy font & voiceless plosives angular font & sonorants


Congruent associations are expected to induce faster reaction times than incongruent associations for each 2×2 interaction of parameters under study.

Given Cuskley et al.’s results, we could further expect an interaction between the type of frames and the font, with again faster response times in congruent situations than in incongruent situations (see (b) in Table 2).

The hypothesis of an interaction between font and phonetic composition could finally be made, considering sound symbolic associations with letter shapes in a similar fashion as with frames. Once again, faster response times were expected in congruent situations than in incongruent situations (see (c) in Table 2). As explained above, response times in the case of voiced plosives were difficult to predict since these consonants could be congruent with spiky frames because they were plosives, or congruent with rounded frames because they were voiced. Which association would be stronger could not be anticipated, and we thus did not have specific hypotheses.

This experimental design thus allowed us to test three hypotheses of interaction. Given the previous studies and assuming the existence of simultaneous effects, we postulated that the three preceding interactions could be significant. Finally, we did not have expectation about a potential triple interaction *Type of frames × Category of consonants × Font*.

### Participants

21 female and 20 male students from universities in Lyon, aged from 18 to 30 years (average 22.2 years), were recruited (N = 41). All were monolingual native French speakers and had a normal vision or corrected to normal, with no history of speech or hearing disorders reported at the time of experiment. Six of them were left-handed.

### Material

#### Words and pseudowords

We defined a number of criteria to select words and create pseudowords. All strings (i.e. both words and pseudowords) contained:

- three, four or five letters;
- three or four phonemes;
- three possible syllabic structures: CVC, CVCV or VCVC (C stands for consonant, V for vowel).

Specific constraints were applied to the choice of vowels, as detailed in S1 Protocol.

We collected 233 words corresponding to our criteria, with associated information in the *Lexique 3*.*81* database [24]:

- written and oral frequencies (respectively in books and in movies);
- number of letters and phonemes;
- syllabic structures.

We further extracted the categories of consonants: word made of plosives, of sonorants or mixed.

In parallel, we generated 974 pseudowords. Apart from frequencies, similar information as for words was compiled.

For both words and pseudowords, orthographic and phonological neighbors were figured out on the basis of Luce and Pisoni [25]’s method by deleting, adding or substituting one phoneme / letter (for phonological / orthographic neighbors) in any position. Once neighbors were found, neighborhood frequencies were computed.

On the basis of the preceding corpora, a genetic-algorithm-based program named *Bali* [26] was used to generate lists of words and pseudowords that were as balanced as possible with respect to confounding variables (number of letters, of phonemes, of phonological and orthographic neighbors, frequencies of these neighbors etc.), and as internally diverse as possible. This was in order to produce a well-balanced corpus and a variety of combinations for later regression analyses.

Lists of pseudowords were first generated, then lists of words were created with lists of pseudowords as counterparts in the balancing optimization process.

For pseudowords, four lists were created: one with voiced plosives, one with voiceless plosives, and two with sonorants–in order to have as many pseudowords composed of sonorants as of plosives. For words, four lists were also created: one with plosive-only words, one with sonorant-only words, and two with mixed words.

We obtained a total of 256 character strings, divided into 128 words and 128 pseudowords. Half (64) of the pseudowords were composed of sonorants ([l, m, n]), half of plosives. Furthermore, half of the latter (32) were composed of voiceless plosives ([p, t, k]), and half (32) of voiced plosives ([b, d, g]). In the group of words, 32 were composed of sonorants, 32 of plosives (voiced or voiceless) and 64 were mixed words (containing both sonorants and plosives, whatever the voicing) (see Table 3, and see S1, S2, S3 and S4 Tables for the actual lists of items and their properties).

**Table 3:** Number of words and pseudowords for each category of consonants in the experimental material.

Words			Pseudowords		
Mixed	Sonorants	Plosives	Sonorants	Plosives	
				Voiced	Voiceless
64	32	32	64	32	32

There were five pairs of homophones among the 128 pseudowords (imale/immal; nalle/nal; lummu/lumue; lul/lulle; nanu/nannu), and one among the 128 words (laque/lac), with no occurrence of two homophones in the same list.

#### Frames

**Fig 1:**
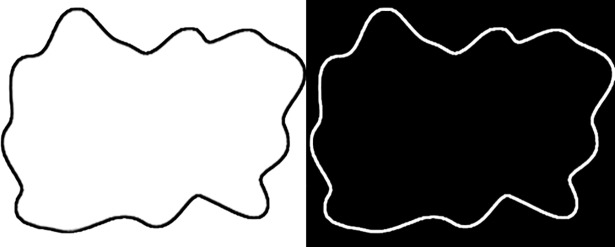
Original frame used in Westbury [9]’s experiment (left) and corresponding edited frame in our experiment (right).

In Westbury’s original experiment, frames were presented as white objects on a black background. Yet, to avoid eye strain due to the presentation of character strings on a white background (in the center of the frame), we decided to keep only the contours of frames, presented in white on a black background (see Fig 1).

**Fig 2:**
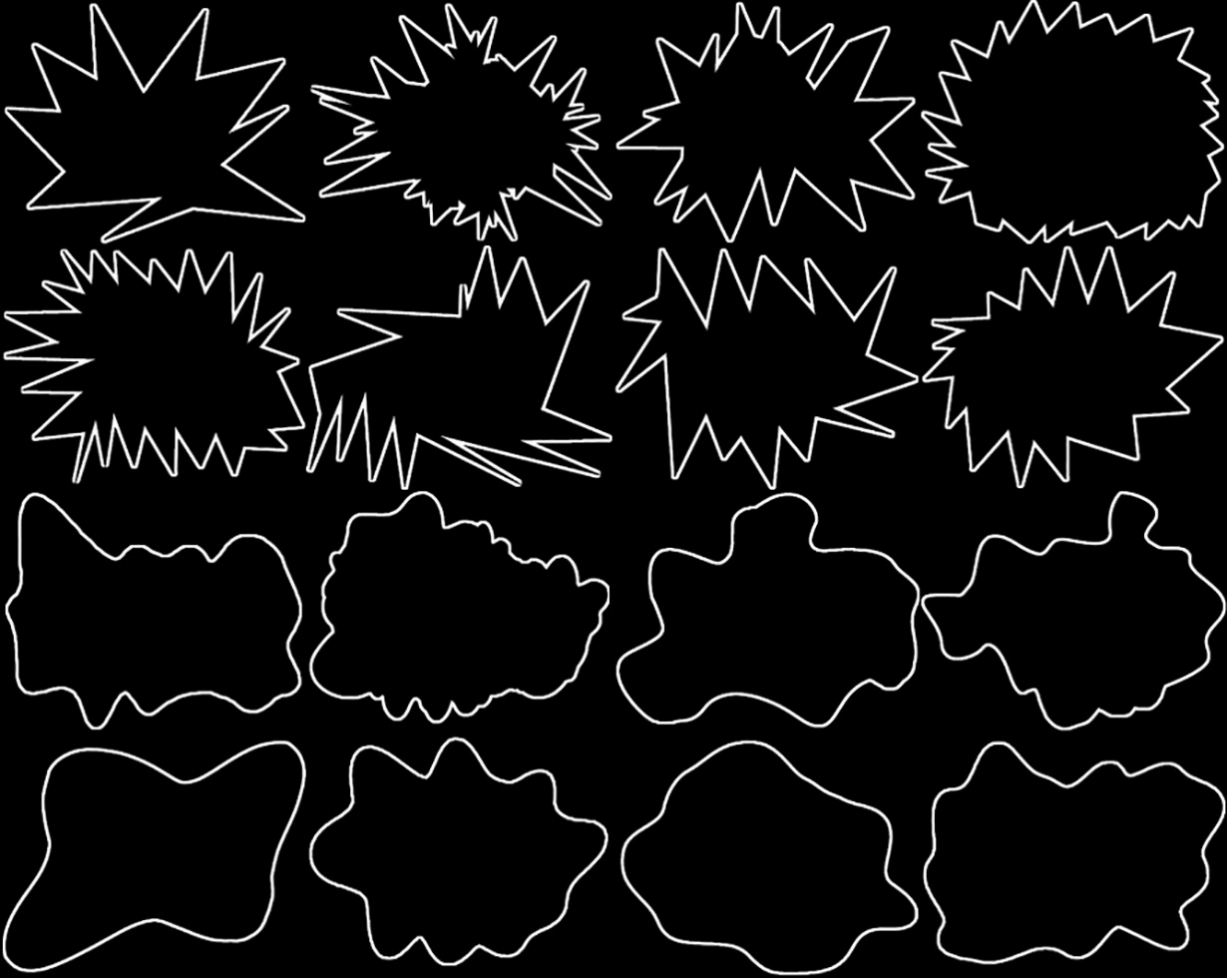
The 16 frames used in the experiment. The top eight frames are considered spiky, the lower eight rounded.

We selected 16 of the 40 frames used in Westbury’s experiment–eight spiky and eight rounded–in order to focus on those that seemed most relevant to assess sound symbolic effects. To this end, we chose shapes that were as asymmetric, unambiguous in terms of roundedness or spikiness, and non-reminiscent of existing or imaginary entities (like ghosts), as possible (see Fig 2).

#### Fonts

**Fig 3:**
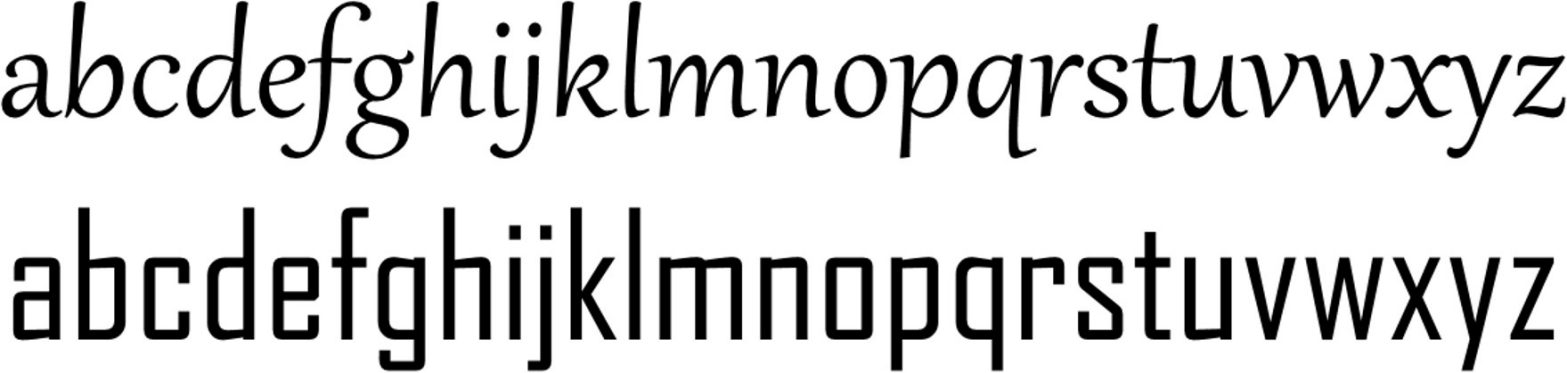
The *Agency FB* and *Gabriola* fonts used in the experiment. The *Agency FB* font (bottom) is the angular font, and the *Gabriola* font (top) the curvy font.

**Fig 4:**
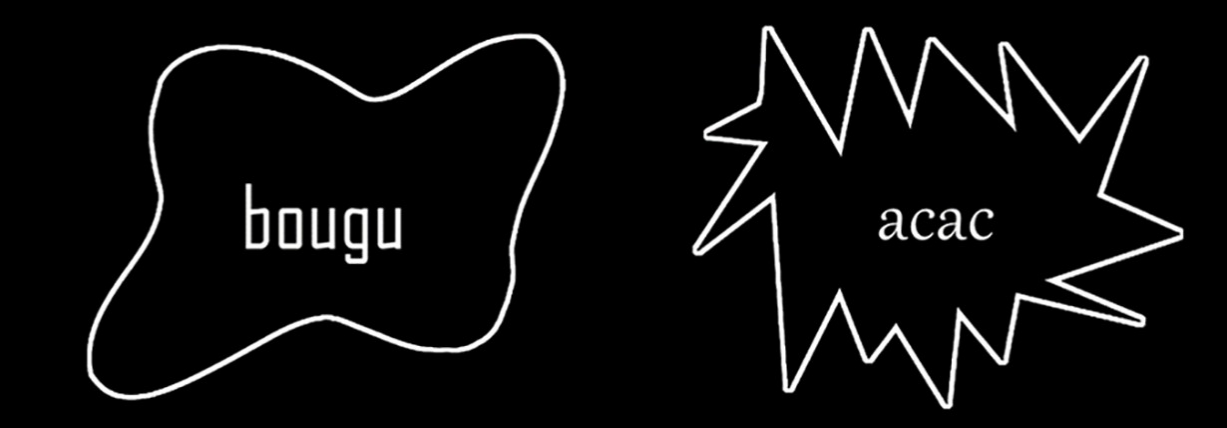
Examples of trials with two pseudowords. On the left, the pseudoword is presented in a round frame with the angular font, on the right in a spiky frame with the curvy font (Gabriola).

*Agency FB* was chosen as our ‘angular’ font due to its right-angled letters. *Gabriola* offered rounded letters without right-angled corners, and was therefore chosen as our ‘curvy’ font (see Fig 3). No formal test was, however, performed, or judgment task conducted, as for the angularity or curviness of the letters. Fig 4 offers two examples of written forms displayed in a frame, as presented to participants.

### Procedure

The open-source software *OpenSesame* [27] was used to generate the experiment and collect answers and response times, more specifically with the *psycho* back-end, which relies on *PsychoPy* and offers good temporal resolution for display and response time.

Subjects entered their choice—word or not—with two keys (see S1 Protocol). They were asked to answer accurately and as fast as possible.

A fixation point was presented for 200 ms, then the frame appeared for a varying duration (between one and three seconds) before the character string appeared in its center (this corresponds to the stimulus-onset asynchrony or SOA). The string and the frame were displayed until the answer of the participant, otherwise they disappeared after 2 seconds. Then, a mask composed of a succession of three images of a Gaussian noise was presented for 99 ms (33 ms for each image) to avoid any retinal persistence.

The experiment began with a training phase in which height trials were presented (four words and four pseudowords). These practice stimuli were not presented again in the main experiment. After the training, the percentage of success was displayed on screen to both the participant and the experimenter, which allowed the latter to ensure the former understood the instructions and used the right keys. The experiment was then divided in four blocks of 64 trials interspersed by breaks whose duration was determined by the participants themselves.

The matchings (between the phonetic categories related to the character strings, the type of frame and the font) were generated in a pseudorandom way for each subject. Half of the pseudowords were presented in a spiky frame, and half in a rounded frame. Half of the pseudowords in each of these two conditions were displayed with the *Gabriola* font, and half with the *Agency FB* font. Finally, the category of consonants of the pseudoword was taken into account: each match between a type of frame and a font (for example, spiky and angular) was presented with 16 sonorants, eight voiceless plosives and eight voiced plosives (see Table 4).

**Table 4:** Distribution of experimental stimuli with respect to type of frame, font and category of consonants.

Spiky frame						Round frame					
*Gabriola*			*Agency FB*			*Gabriola*			*Agency FB*		
Sonorants	Plosives		Sonorants	Plosives		Sonorants	Plosives		Sonorants	Plosives	
	Vd	Vs		Vd	Vs		Vd	Vs		Vd	Vs
16	8	8	16	8	8	16	8	8	16	8	8

Vd stands for Voiced, Vs for Voiceless.

The order of presentation of the stimuli was constrained to avoid repetition effects (see S1 Protocol).

## Results

For all statistical analyses, we used the R project [28] with especially the package ggplot2 for graphics [29], reshape and plyr [30] for data manipulation, and lme4 [31] and gamlss [32] for generalized mixed modelling.

### Success rate

Following Westbury [9], we chose to a priori eliminate subjects who had more than 20% of erroneous answers. The highest error rate was 12.9%, hence all 41 subjects were taken into account.

### Presentation of the response times

Only correct responses were selected for further analysis. For these responses, the average response time was then 848 ms (sd = 243ms) for pseudowords, and 810 ms (sd = 246 ms) for words. There was no trimming of the data due to the skewness of the distribution of response times, both for words and pseudowords (see S1 Analysis). The datasets for pseudowords and words are available as supplementary material (see S1 Dataset and S2 Dataset respectively, as well as S1 Structure for a detailed description of their content).

### Analysis of response times for pseudowords

Regarding explanatory variables, we included the font, the type of frame and the category of consonant, as well as their three two-by-two interactions and their triple interaction. We also included the trial position and the response time of the preceding trial, as justified in S1 Analysis.

The fixed effects were therefore:

- **Font** (Angular or Curvy)
- **Type of Frame** (Spiky or Round)
- **Category of consonants** (Voiceless Plosives, Voiced Plosives or Sonorants)
- **Type of Frame × Font**
- **Category of consonants × Font**
- **Type of Frame × Category of consonants**
- **Type of Frame × Font × Category of consonants**
- **Trial position**
- **Preceding Response Time**

We considered three random effects to account for the non-independence of our response times and to avoid any type of pseudo-replication [33]:

- **Subject** (for our 41 participants)
- **Stimulus** (for the 128 pseudowords)
- **Frame** (for our 16 frames)

Other variables such as the number of letters, the syllabic structure etc. could have been included as predictors in the model too, thus adding an a posteriori control to the a priori control (see Section Words and Pseudowords). However, there were then high levels of multicollinearity between the predictors, which violated the assumptions of the models. We hence chose not to include these variables, rather than to complicate the statistical analysis with a selection of the best set of predictors (based on variance inflation factors).

In order to model error terms correctly, we compared different generalized mixed-effect regression models with response time as dependent variable. To do so, we initially relied on models with distributions belonging to the so-called exponential family, as made available by the ***glmer()*** function of the lme4 package. We then switched to generalized additive models for location, scale and shape (GAMLSS) [32,34,35], available in the gamlss package. Details of why and how we compared these models are given in S1 Analysis.

We found that the *Generalized Gamma* (GG) distribution, which is a generalization of the *Gamma* (GA) and *inverse Gaussian* (IG) distributions, was an appropriate choice for error terms. Only the location parameter of the distribution was modelled with the previous predictors, other parameters of scale and shape were estimated by an intercept only. While location corresponds to the mean in *inverse Gaussian* and *Gamma* distributions, it does not in the *Generalized Gamma*. It was, however, proportional to it given that scale and shape were modelled by intercepts only in our approach.

While we report below the outputs of GG models, we also computed results for other distributions in order to assess effects over a range of models, and therefore increase our confidence in them.

A first model was run on the whole set of pseudowords (n = 5,100). Observations corresponding to normalized quantile residuals below -2.5 or above 2.5 were removed (see S1 Analysis), and the model was updated on the trimmed dataset (n = 5,035) before further computations were performed. This strategy, suggested by Baayen and Milin [36] and named model criticism, was preferred to a−priori trimming, since it better accounted for the specific, non-Gaussian, distribution of error terms of each generalized regression model. Assessments of the assumptions underlying the model were all satisfactory (see S1 Analysis).

The significance of the predictors was assessed with Likelihood ratio tests (LRT). The triple interaction was non-significant (***Δ AIC*** = 4, ***Df*** = 2, ***LRT*** = 0.015, ***p*** = 0.99), and double interactions were assessed once it was removed from the model.

Results are given in Table 5. The first column indicates which predictor term is dropped in the nested model. Except for the full model, the second column (Df) gives the difference of degrees of freedom between the full model and the nested model. The fourth column (LRT) reports the difference in deviance between these two models, and the fourth column (Pr(Chi)) the *p*-value of the χ^2^ test on the difference of deviance. **Type of Frame**, **Category of Consonant** and **Font** are absent as main effects given the presence of their interactions. *P*-values suggested a significant interaction for **Type of Frame × Font**, but not for the other two interactions. This result was overall congruent with what was found with other distributions (IG, GA, Johnson’s SU, see S1 Analysis).

**Table 5:** Likelihood ratio tests for the fixed predictors of response times for pseudowords in a Generalized Gamma gamlss model.

	Df	AIC	LRT	Pr(Chi)
Full model		64,715		
**Type of Frame : Cat. Of Consonant**	2	67,711	0.75	0.689
**Type of Frame : Font**	1	64,719	6.84	0.009
**Cat. Of Consonant : Font**	2	64,714	3.02	0.221
**Trial position**	1	64,820	107.84	< 0.001
**Preceding response time**	1	64,816	103.20	< 0.001
**Subject (random)**	40.5	66,524	1,890.92	< 0.001
**Stimulus (random)**	114.4	65,419	933.42	< 0.001
**Frame (random)**	1.1	64,714	2.17	0.161

Df stands for ‘degrees of freedom’, AIC for ‘Aikake Information Criterion’, and LRT for ‘Likelihood ratio tests’.

**Fig 5:**
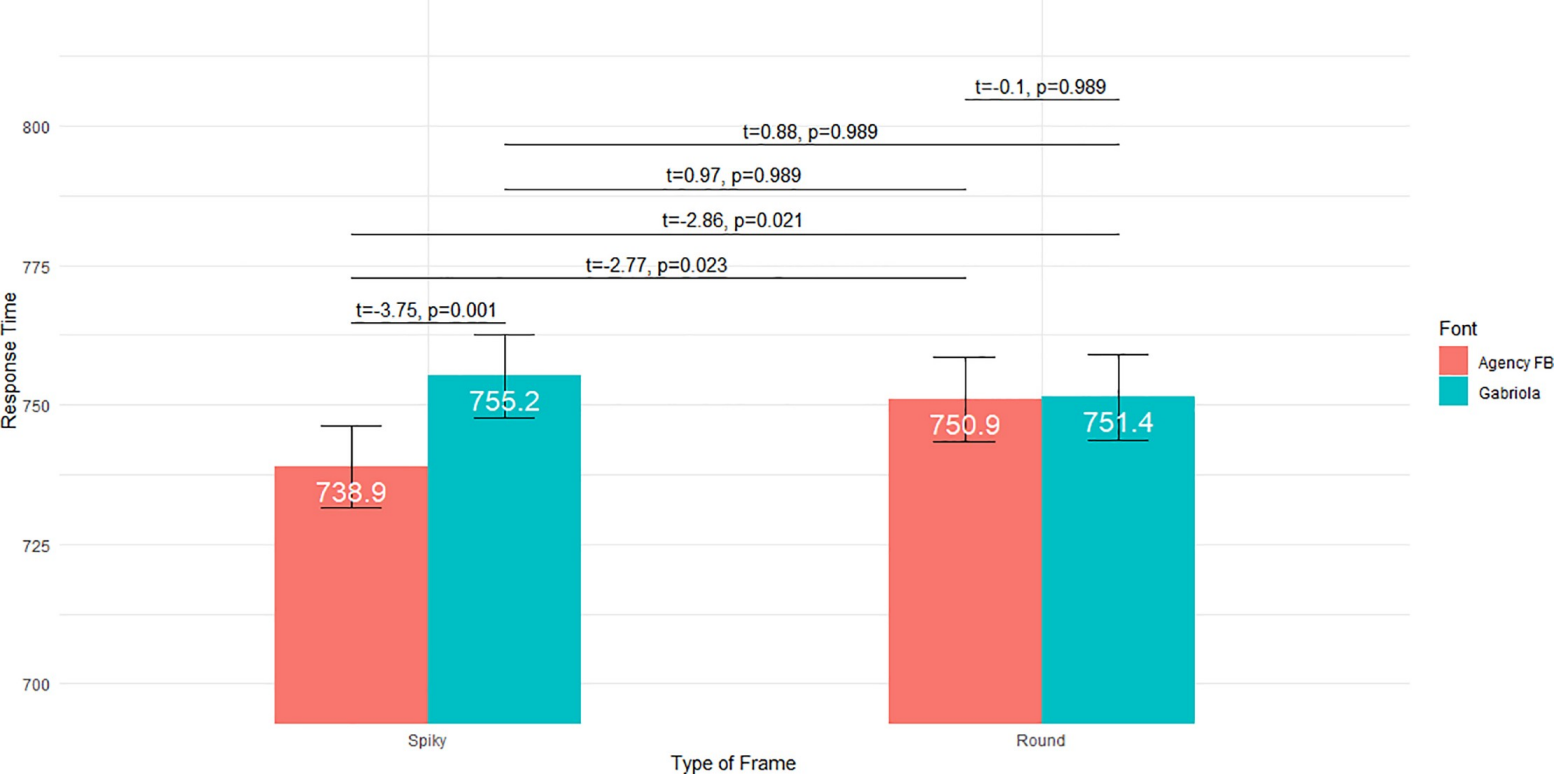
Interaction between Type of Frame and Font for pseudowords. Marginal locations are displayed numerically (white figures) and graphically for the four conditions *Spiky Frames* & *Angular Font*, *Spiky Frames* & *Curvy Font*, *Round Frames* & *Angular Font* and *Round Frames* & *Curvy Font*. Vertical bars report the confidence intervals for the four means. Significance levels of pairwise comparisons of these conditions are reported above. *P*-values have been corrected for multiple comparisons with Holm’s method.

To further understand the pattern of interaction between the type of frame and the font, we assessed the six possible contrasts between the four conditions ***Spiky Frames* & *Angular Font***, ***Spiky Frames* & *Curvy Font***, ***Round Frames* & *Angular Font*** and ***Round Frames* & *Curvy Font***. We first computed the estimated marginal locations of the response times in the four conditions, i.e. the locations adjusted for other variables in the regression models. For each contrast between two marginal locations, a z-test of the difference between these locations was then performed. We considered the Holm correction to decide which null hypotheses should be rejected when controlling for the inflated type I error rate due to multiple comparisons [37]. Fig 5 summarizes the values of the four marginal locations and the results of the six z-tests of difference.

The *a priori* congruent ***Round Frames* & *Curvy Font*** condition does not induce faster response times than the *a priori* incongruent conditions ***Round Frames* & *Angular Font*** and ***Spiky Frames* & *Curvy Font***. On the contrary, the *a priori* congruent ***Spiky Frames* & *Angular Font*** condition is faster than the two corresponding incongruent conditions, and also than the ***Round Frames* & *Curvy Font*** condition. Overall, the interaction is therefore due to the faster response times obtained in the ***Spiky Frames* & *Angular Font***, compared to the three other conditions.

### Analysis of response times for words

We applied the same analytical procedure to words. Once again, a GG distribution appeared appropriate with respect to error terms.

In the initial model with all entries (n = 4,570), 43 entries had normalized quantile residuals higher than 2.5 or lower than -2.5, and were discarded in a second model (n = 4,527). Assessments of the assumptions underlying the model were all satisfactory.

Once again, the triple interaction **Type of Frame × Font × Category of Consonant** was non-significant (***Δ AIC*** = 1, ***Df*** = 3, ***LRT*** = 4.34, ***p*** = 0.23), and double interactions were assessed once it was removed from the model. Table 6 reports the various LRT performed.

**Table 6:** Likelihood ratio tests for the fixed predictors of response times for words in a Generalized Gamma gamlss model.

	Df	AIC	LRT	Pr(Chi)
Full model		58,349		
**Type of Frame : Cat. Of Consonant**	3	58,351	8.14	0.043
**Type of Frame : Font**	1	58,352	5.50	0.019
**Cat. Of Consonant : Font**	3	58,347	4.30	0.231
**Trial position**	1	58,348	107.84	0.373
**Preceding response time**	1	58,385	103.20	< 0.001
**Subject (random)**	40.2	59,630	1,890.92	< 0.001
**Stimulus (random)**	117.2	59,097	933.42	< 0.001
**Frame (random)**	0.0	58,349	0.00	< 0.001

Df stands for ‘degrees of freedom’, AIC for ‘Aikake Information Criterion’, and LRT for ‘Likelihood ratio tests’.

**Fig 6:**
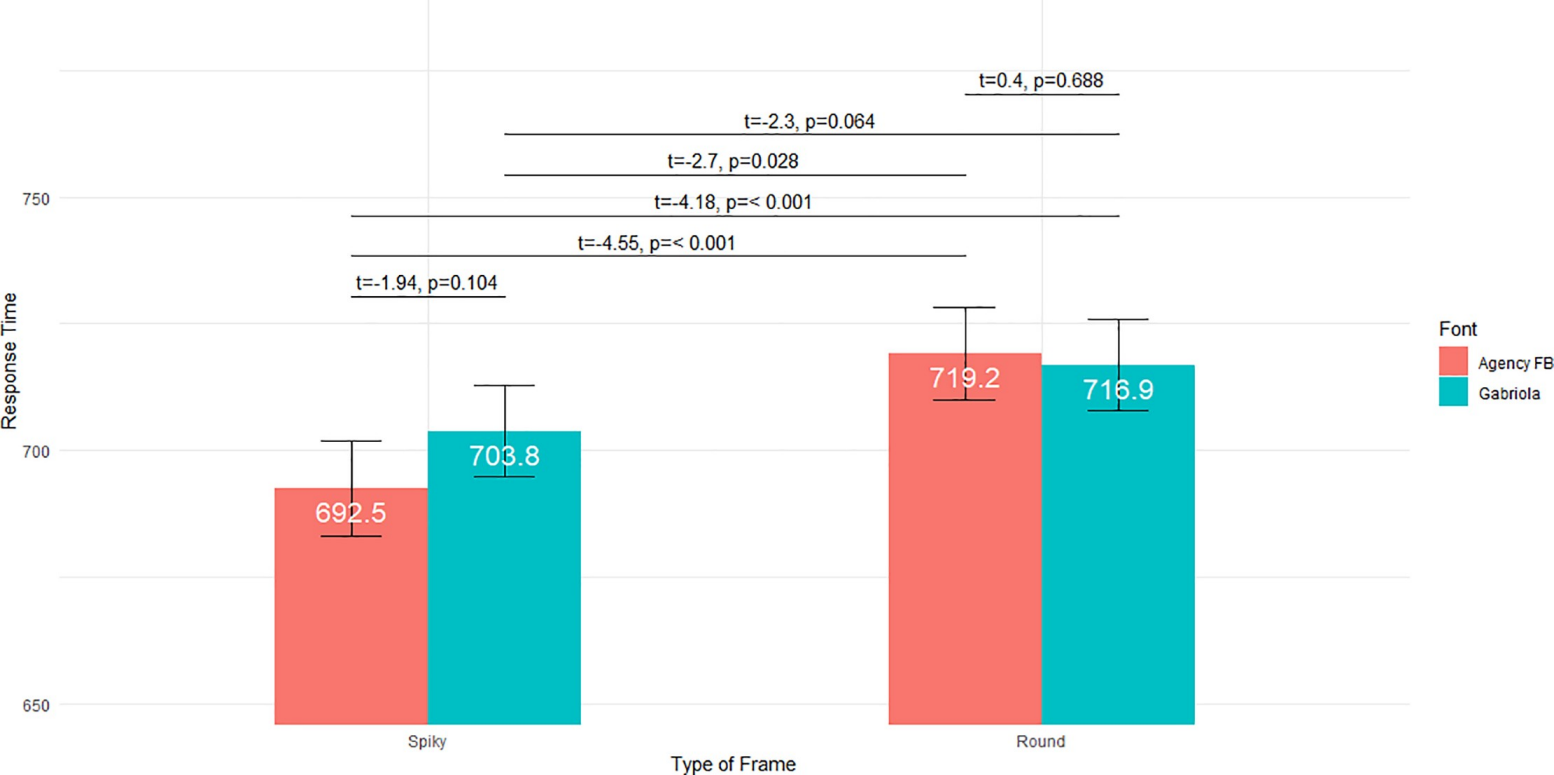
Interaction between Type of Frame and Font for words. Marginal locations are displayed numerically (white figures) and graphically for the four conditions *Spiky Frames* & *Angular Font*, *Spiky Frames* & *Curvy Font*, *Round Frames* & *Angular Font* and *Round Frames* & *Curvy Font*. Vertical bars report the confidence intervals for the four locations. Significance levels of pairwise comparisons of these conditions are reported above. *P*-values have been corrected for multiple comparisons with Holm’s method.

*P*-values suggested a significant **Type of Frame × Font** interaction, no significant interaction for **Font × Category of Consonant**, and a **Type of Frame × Category of Consonant** interaction. Regarding **Type of Frame × Font**, computations of the marginal locations and of their contrasts are given in Fig 6. The pattern was reminiscent of what was observed for pseudowords. However the difference between ***Spiky & Agency FB*** and ***Spiky & Gabriola*** was at the 0.05 level before the Holm correction, and higher after. Rather than ***Spiky & Agency FB*** being significantly different from the three other conditions, the model therefore suggested a main effect of **Type of Frame**, with shorter response times for spiky frames than for round frames. Once again, models with different distributions (IG, GA, Johnson’s SU) gave similar results qualitatively, although the significance of *p*-values varied from one model to the next. The JSU model in particular suggested both a main effect of **Type of Frame**, with shorter response times for spiky frames, and, as for pseudowords, shorter response times for ***Spiky & Agency FB*** compared to the three other conditions.

**Fig 7:**
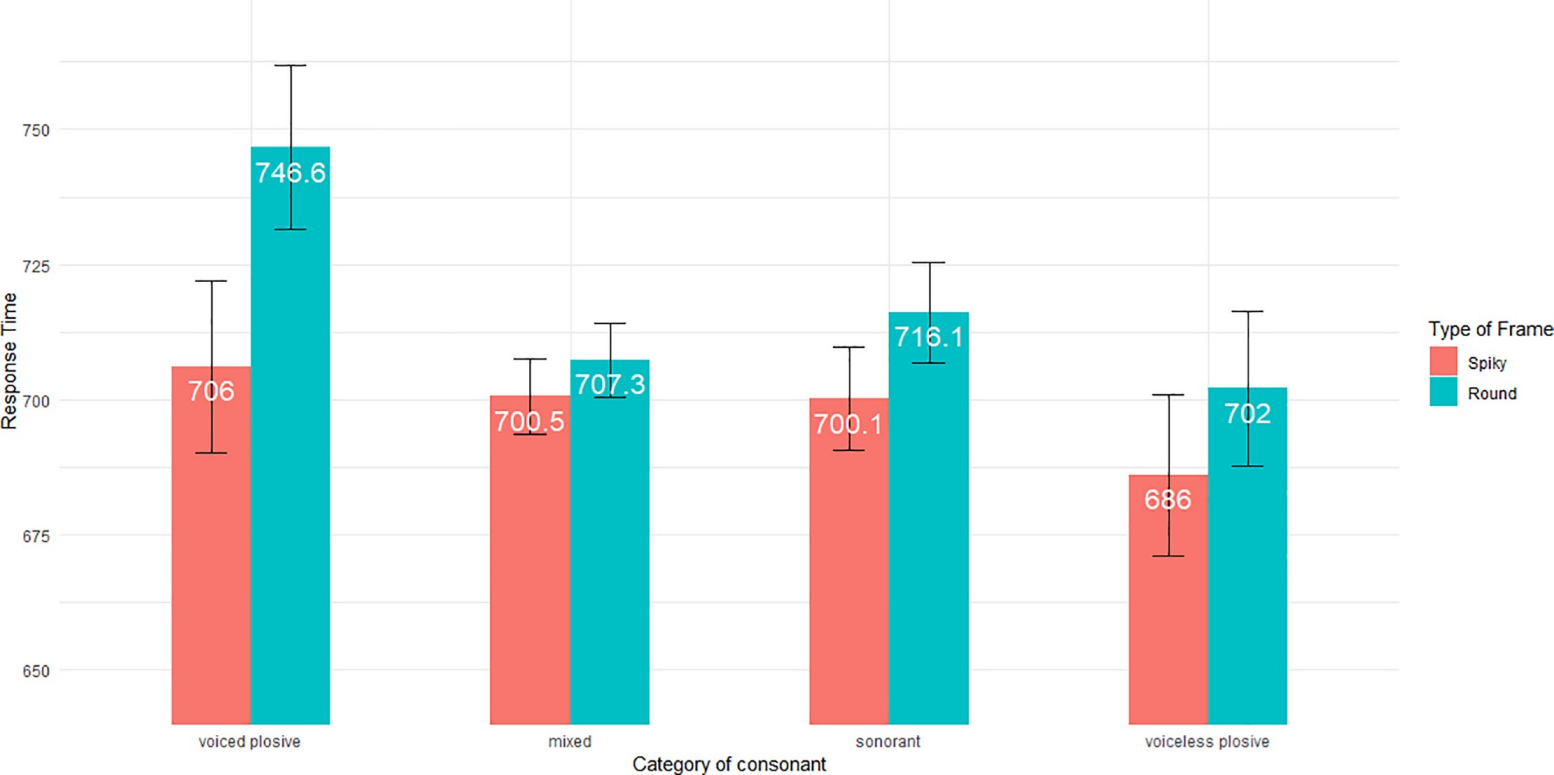
Interaction between Type of Frame and Category of Consonant for words. Marginal locations are displayed numerically (white figures) and graphically for height different conditions. Vertical bars report the confidence intervals for the four locations.

As for the **Type of Frame × Category of Consonant** interaction (Fig 7), the pattern of response times ran counter to our hypothesis (a), since for example round frames and sonorants led to longer response times than spiky frames and sonorants. This interaction was thus unsupportive of sound symbolism. What can be stressed is the case of voiced plosives, with in particular much longer response times for the **Round × Voiced Plosive** condition, compared to all other conditions. This effect likely explains why the interaction was significant with LR tests. We had no specific predictions for voiced plosives, and the result for the **Round × Voiced Plosive** condition is difficult to explain. Also, the **Type of Frame × Category of Consonant** interaction was not found when considering IG or GA distributions rather than GG, which casts doubts over its actual significance.

As a summary regarding our three oriented hypotheses, we did not get any interaction between **Font** and **Category of consonants**. In words, but not pseudowords, we found a statistically significant interaction between **Type of Frame** and **Category of consonants**, in conflict with sound symbolism. Finally, we observed a statistically significant interaction between **Type of Frame** and **Font** for both pseudowords and words. More precisely, with pseudowords, we observed faster responses for spiky frames and angular letters than for the three other conditions (spiky frames and curvy letters, round frames and angular letters, and round frames and curvy letters). For words, we saw rather a main effect of spiky frames.

While the amount of difference between the two fonts was not assessed a priori with a pre-test, this result shows that differences were large enough to elicit a differentiated pattern of answers.

### Subjects’ reports about the experiment

Upon finishing the experiment, we asked subjects if they had noticed something special. If they answered yes, we then asked what was special and eventually if they had noticed that there were different fonts. None of the 41 subjects spontaneously reported the existence of two fonts and only 10 subjects answered yes when explicitly asked. One subject reported faster answers when the frame was spiky, and another one when it was spiky and when the word had a negative valence.

## Discussion

We did not observe the cross-modal interaction between phonology and visual form found in Westbury [9]. We obtained faster answers only when the spiky frames and the angular font were displayed together. This brought us closer to Cuskley et al.’s proposal of visual interaction effects, suggesting that such effects should be taken into account while investigating sound symbolism. Below, we focus on the visual processes possibly underlying the specific results we obtained, first with respect to geometric shapes in general, second with respect more specifically to written words and pseudowords. We then discuss the cognitive processes underlying sound symbolism, in relation to the transparency of the task, and in terms of low and high-level processes. We point in particular to the possibility of ideasthetic processes in addition to synaesthetic ones.

### Visual processes

#### Low-level visual processes in tasks involving angular and curvy visual shapes

Faster answers for the combination of spiky frames and angular letters suggest an effect of visual saliency. Indeed, some studies highlight an attentional enhancement due to simple geometric shapes. For example, some minimal stimulus configurations enhance the capture and maintenance of attention [38]: downward-pointing stimuli (a downward pointing V or triangle) are more rapid to detect than other stimuli such as an upward-pointing V or triangle, or a circle. Moreover, this shape induces greater difficulty to disengage attention. This attentional modulation can be explained by a negative valence carried by angular configurations, especially downward-pointing stimuli. Negative stimuli are indeed known to be faster to detect and to retain attention for a longer time than positive stimuli. Armbruster, Suchert, Gärtner and Strobel [39] collected ratings about angular and curvy configurations and found that downward pointing triangles are judged as more negative and arousing than upward pointing triangles, which are in turn more negative and arousing than circles. These assessments are further in line with measures of peripheral physiological parameters. Difference in cognitive processing between upward pointing and downward pointing triangles are further evidenced by fMRI studies [40]

Different authors suggest a connection between geometric shapes and faces: facial features expressing anger are angular and diagonal forms (e.g. frowning eyebrows) including acute angles pointing downward, while happiness is characterized by curved patterns [41]. In particular, Bassili [42] showed that anger is characterized by a downward movement on the forehead due to frowning. Aronoff et al. [41] conducted a study in which masks of several cultures, either threatening or not, were evaluated. Cross-culturally, masks expressing threat contained more triangular eyes or visible teeth than nonthreatening masks. Some features are direct iconic representations of facial expressions (e.g. frowning) but others (e.g. pointed ears) seem to convey a subjective meaning that may derive from basic visual patterns involving two specific features: angularity and diagonality. Coelho, Cloete, & Wallis [43] evaluated in a more systematic way the impact of emotional content using different types of stimuli in a visual search paradigm. Comparing schematic faces with abstract faces built with straight or curved features, they reached the conclusion that subjects’ answers are explained neither by resemblance to faces and the associated emotions, nor by judgments of valency, but rather by the characteristics of lower-level visual cues. The orientation of features seems to be the key parameter regarding differences in detection speed: lines perpendicular to the edge are more rapidly detected, therefore more salient, than concentric features.

With respect to our experiment, the previous studies would suggest a main effect of the font, with faster response times for angular letters, and a main effect of the type of frames, with faster response times for spiky frames. However, we observed a more complex pattern of effects with faster response times for pseudowords only when the sharp angles found in spiky frames co-occurred with those found in angular letters. This interaction suggests that although the visual saliency of such stimuli seems to play a role, it is a component of a more complex cognitive treatment.

Regarding pseudowords, a possible explanation is that while the dissimilarities between spiky/angular and round/curved shapes are not enough in the context of the lexical decision task to induce differences in response times, an asymmetrical priming effect takes place when angular letters are displayed within spiky frames: contrary to round frames, spiky frames first arouse attention to sharp angles and perpendicular lines, which then facilitates the processing of angular letters.

As for words, there was rather a main effect of the type of frame, with spiky frames corresponding to shorter response times, than a specific interaction between angular letters and spiky frames. A possible explanation for this different pattern of results echoes the ideas underlying dual-route models in reading: along a first route, words are processed more holistically, with access to the mental lexicon; unknown written forms–and therefore pseudowords–are deciphered along a second route on the basis of grapheme-phoneme association rules [22]. Along these lines, the processing of written words could be less impacted by graphemic features than the processing of written pseudowords. There would be therefore no priming effect of spiky frames on angular letters.

#### From processing geometric shapes to processing written pseudowords

Beyond these interpretations in terms of basic geometric features, an alternative or rather complementary explanation lies in the processing of written pseudowords in terms of linguistic stimuli, and not as arbitrary assemblages of basic shapes.

According to Dehaene and Cohen [44], an area localized in the left fusiform gyrus, in the visual occipital-temporal stream, appears to respond more to words than to other visual objects: the visual word form area, or VWFA. In Baker et al. [45] for example, English words and strings of consonants elicit stronger responses in English speakers than line drawings of things, numbers, or characters from another writing script (Hebrew letters and Chinese ideograms). As stated in Dehaene and Cohen [44], the VWFA would result from ‘*a putative mechanism by which a novel cultural object encroaches onto a pre-existing brain system*’, in agreement with Dehaene [46]’s ‘neuronal recycling’ hypothesis. In other words, the VWFA would thus develop ontogenetically in preadapted brain areas to process the specific patterns of written linguistic stimuli. In underpinning their proposal, Dehaene and Cohen mention that the frequencies of intersections in writing systems follow a universal and natural frequency distribution, i.e. similar to what is found in natural images [47]. Hence, writing systems seem to follow rules enacted by more general visual capacities. Their treatment in the VWFA would therefore be an exaptation of an initial bias in favor of the recognition and treatment of geometric features that are close to those used in letter shapes: line junctions, by which an object occludes another. This is supported by the fact that the area analogous to VWFA in primates encodes intersections [44].

Szwed et al. [48] have underlined the primary role of line junctions. They investigated brain activations when perceiving objects and words while preserving either vertices or ‘midsegments’ in their drawing. For both objects and words, it appears that recognition relies predominantly on line vertices, i.e. where line junctions occur. Activations following the display of stimuli with preserved vertices partially overlap the fusiform gyrus, which is involved in reading, even if it does not imply the VWFA directly.

As recalled by Newman and Twieg [49], a number of word reading studies have shown that ‘*pseudoword and real word reading tended to activate the same cortical network and that pseudoword reading is more effortful*, *producing more activation than real word reading*’ (p. 39). The VWFA falls into such brain areas, with greater activations for pseudowords than for words. This suggests an implication of this area in a prelexical rather than lexical process [50]. A potential confounding factor is, however, that, as indicated by these authors, pseudowords are also accompanied by slower responses and longer activations. The greater BOLD (blood-oxygen-level dependent) signal observed in fMRI studies may therefore be due to a longer activation, and not a stronger one.

Although the VWFA does not respond to non-linguistic stimuli, Szwed et al. [48] showed that the vision of line junctions activates close neuronal structures in the fusiform gyrus. The spreading of activation to the VWFA that could follow is the possible neuronal basis for the asymmetrical priming effect we proposed earlier. Additionally, the frames used in our study did not result from a random placement of dots and either straight or curved lines as in Nielsen & Rendall [6,14] or in Monaghan et al. [16]. There could therefore be a bias due to the experimenters’ involvement in the design of the frames, with features reminiscent of those coded by the fusiform gyrus or even the VWFA.

### Transparency of the task and cognitive level of response

#### Implicit vs explicit protocols

While many studies have highlighted the existence of the bouba-kiki effect, our results did not. A possible explanation is that the implicit nature of our protocol explains the discrepancy with results from association tasks of other experiments. As already explained, tasks which do not explicitly ask the subjects to make associations dissimulate the phonetic and visual contrasts to a greater extent. One can reasonably admit that protocols can be evaluated along a continuum with respect to the transparency of their task. In other words, transparency is not a yes-or-no property. Along such a continuum, our protocol stands as rather opaque compared to others, which would explain the absence of sound symbolic effects.

Less transparent does not mean, however, that participants do not engage in metacognitive reasoning about the task. In our experiment, subjects were asked to perform a lexical decision task, without any reference to the frames or the fonts. Although metacognitive strategies may have taken place regarding the frames, we argue that the differences between the angular and curvy fonts were much less noticed, especially since none of our 41 subjects spontaneously reported that two different fonts were being used.

The discrepancy between our results and Westbury [9]’s remains to be accounted for, since our protocol derived from his and shared his implicitness. A first possibility lies in differences in terms of statistical approaches. In particular, the issue of non-independence was only partially addressed with the by-item and by-subject approaches used by Westbury. Another explanation relates to differences in controlling for the potential confounding factors (number of phonological/orthographic neighbors, preceding response time etc.). The difficulty of our task may be another reason: the contrasted graphemes of our two different fonts could have worked as a cognitive distractor and masked an intrinsically weak sound symbolic effect. Actually, our response times seem to be quite long for a lexical decision task (810 ms for words and 848 ms for pseudowords). For the sake of comparison, response times for a lexical decision task in French [51] are respectively 730 and 802 ms. We argue, however, that these differences are not due to a greater difficulty of our task because of a lower readability of the fonts. On the one hand, the readability seemed to be equivalent for both fonts, since there was no main effect of the **Font** variable. On the other hand, response times were trimmed in Ferrand et al. [51]’s study, but not in ours. Given the likely right-skewed distribution of response times in the former, this likely explains the differences in mean response times.

Overall, our results support Nielsen and Rendall [14]’s argument that the strength of the bouba-kiki effect is related to the transparency of the testing protocols. In a very opaque procedure, sound symbolic associations, if they exist, may be too weak to be revealed statistically, even with a large number of observations.

#### Arguments in favor of low-level processes

What are the cognitive processes at play in sound symbolism, and were they underlying our subjects’ answers despite the lack of significant sound symbolic interactions? More precisely, what is the ‘level’ of these processes?

A number of studies are in favor of low-level processes, which occur early and automatically in the processing of stimuli. Vainio, Tiainen, Tiippana, Rantala and Vainio [52] conducted experiments in which subjects were presented with objects differing on two dimensions–shape and size–and requested to produce isolated syllables or vowels according to one of the two preceding dimensions. The effect of the second dimension, which was not relevant to the task, was studied. The authors demonstrated that a spiky shape shortened the reaction time for the vocalization of /i/, /ti/ or /te/, *mostly* when participants correctly categorized the visual stimulus as little. Conversely, a round shape shortened the reaction time for the vocalization of /mɑ/, /me/ and /u/ *only* when participants correctly categorized the visual stimulus as big. These results supported correspondences between articulatory movements and visual features, and demonstrated an implicit impact of a non-relevant modality (shape) on a size-categorization task via an articulatory medium response.

A couple of studies suggest that sound symbolic associations can be detected in early neurophysiological processes. Kovic, Plunkett and Westermann [53] used a paradigm that consisted first in learning labels for two kinds of ‘animals’–several exemplars of spiky and round creatures. Labels were either congruent or incongruent with the shape of the animals. In a second task, the four possible types of pairs were presented separately and subjects had to decide which pairs were correct according to the rules they had learned. Participants in the congruent condition responded quicker to congruent pairs than to incongruent ones, while participants in the incongruent condition were slower to reject congruent pairs than to reject incongruent ones. This revealed a bias in favor of sound symbolic pairs, regardless of the learning targets. This behavioral result was replicated in a setting with an ERP recording. A negative wave was found to appear between 140 and 180 ms in occipital regions for congruent pairs, which may indicate multimodal integration.

Asano et al. [54] also found cues of multimodal integration in 11-month-old infants which were presented with different audio-visual bouba-kiki associations. This was suggested by the increase, for congruent trials and between 1 and 300 ms, in the amplitude of oscillations recorded in centro-parietal regions. Additionally, a wave corresponding to N400 in adults–a well-known marker of semantic or conceptual incongruity–was found in central regions for incongruent pairs.

The previous results, and in particular the precocity of the brain activations, raise the question of the underlying physiological and psychological mechanisms for cross-modal correspondences. Spence [55] reviews various proposals, and cites Ramachandran and Hubbard [56]’s proposal that sound symbolic associations are explained by a low-level binding between visual and auditory representations, an instance of the more general phenomenon known as synaesthesia, which links sensory representations belonging to different modalities.

An argument in favor of synaesthesia is the possibility for 4-month infants to consistently map particular linguistic stimuli to particular shapes [7]. Chen, Huang, Woods & Spence [3] also explain differences in bouba-kiki associations between easterners and westerners by synaesthesia and underlying differences in perceptual experience.

Overall, there is thus a strong line of arguments in favor of low-level cognitive processes for sound symbolism, such as the building of low-level connections between sensory domains. One may then wonder why we did not observe significant sound symbolic associations in our study. Indeed, while higher-level processes could be affected by a non-transparent protocol and a time-controlled task, this should not be the case for lower-level ones, which take place during the early stages of the cognitive processing. This contradiction suggests that other processes may be at play in the case of written stimuli.

#### Synaesthesia, ideasthesia and the specific case of written representations of speech sounds

There are arguments against the previous explanations of sound symbolism in terms of synaesthesia. First, results in very young infants are debated, with experiments failing to reproduce effects found previously in similar populations [57]. Second, what is referred to as different perceptual experiences in Chen et al.’s study could well be different conceptual derivations from the same sensations, because of different cultural experiences and exposures as a whole. Third, some authors have questioned whether the inducer of a synaesthetic relationship belongs to the sensory or to the conceptual domain [58,59]. For example, in time-unit synaesthesia, in which inducers such as weekdays or months are associated with concurrents such as colors, time units are concepts without direct sensory inputs. In grapheme-color synaesthesia, it has also been shown that the assumed meaning of an ambiguous grapheme is what determines the associated synaesthetic colors [60]. Hence, in situations where concepts rather than sensory representations induce sensory activations, the term ‘ideasthesia’ could be more appropriate than ‘synaesthesia’ [59]. The latter would then be restricted to situations where only sensory representations are involved. In some cases, ‘true’ synaesthesia may therefore not be the right explanation for sound symbolic associations, as suggested below.

While we do not argue against synaesthesia in most cases of sound symbolism, we argue that the use of written words or pseudowords, instead of oral inputs, may actually rather constitute a case for ideasthesia, with its own specific features. Indeed, rather than directly accessing a phonetic form upon hearing an acoustic signal, reading linguistic units implies that a sound representation be reconstructed, in the case of pseudowords, or accessed, in the case of words stored in the subject’s mental lexicon. This is reminiscent of the case of a conceptual rather than sensory inducer of a synaesthetic relationship with visual shapes, in the bouba-kiki case at least. This is true especially if ones consider internal representations of words or pseudowords to be made of phonemes rather than of phonetic units. Phonemes are indeed based on contrasts, and are therefore to some extent more abstract than acoustic representations–‘abstract’ is here a better characterization than ‘conceptual’.

Such abstract contrastive representations may benefit, or perhaps require, explicit contrasts in order for their phonetic referents to engage in sound symbolic associations. In other words, presentation of two pseudowords or of two words differing along one or a few phonetic dimensions could help to emphasize the phonetic units to be matched by visual representations. In our own study, given the absence of linguistic contrasts–only one pseudoword or word was displayed at a given time–, sound symbolic associations may have been harder to trigger. The time limit to answer during a trial also perhaps prevented some associations that could have developed in the longer run with additional cognitive processing. This could hence explain why we did not see significant sound symbolic effects, while they can be observed in more explicit association tasks implying written linguistic material.

There is a priori no reason to mutually exclude low-level synaesthetic processes and higher-level ideasthetic ones, although how they occur simultaneously is an open question to us. Whether reinforcing or competing effects may take place is an interesting issue, which study would require carefully designed experimental settings to promote the various processes. A broader perspective would also consist in going beyond the opposition between low-level sensory processes and higher-level ones, and advocate for an embodied perspective on sound symbolism, where semiotic processes emerge from sensory representations without the unraveling of an abstracting process.

## Conclusion

Our investigation, with a large corpus of data, well-balanced lists of stimuli and rigorous statistical analyses, fails to support sound symbolic associations that we were initially expecting on the basis of previous bouba-kiki studies. Rather, we observed at the visual level the possible consequence of interactions between angular shapes in frames and in letters, but not between round shapes and curvy letters. Beyond explanations of this phenomenon, different conclusions can be drawn regarding sound symbolism.

A first suggestion is that saliency effects and intra-modal correspondences should not be discarded as a possible source of interference when investigating sound symbolism with psycholinguistic experiments. What may appear on the surface as a cross-modal correspondence may indeed turn out to be partly based on phenomena that are not related to sound symbolism. Also, sound symbolic effects may be masked by such phenomena.

A second proposal rests on the existence of different processes leading to sound symbolic associations, with some taking place at a lower level of cognitive processing, for example with crossmodal synaesthetic correspondences, while others rely on more abstract representations and necessitate the right environment to become manifest. This could be the case of ideasthetic processes, especially when written material rather than oral one is involved in the experimental design. Different cases of sound symbolism may thus actually point to differing underlying cognitive processes, and may display different properties upon their respective investigations.

## Supporting information

S1 Table List of pseudowords. (DOCX)

S2 Table Properties of the lists of pseudowords. (DOCX)

S3 Table List of words. (DOCX)

S4 Table Properties of the lists of words. (DOCX)

S1 Dataset Dataset for pseudowords. (XLSX)

S2 Dataset Dataset for words. (XLSX)

S1 Structure Structure of the datasets. (DOCX)

S1 Protocol Additional details of the experimental design. (DOCX)

S1 Analysis Details of the statistical analysis. (DOCX)
