# Supplementary material for: Assessing sound symbolism: Investigating phonetic forms, visual shapes and letter fonts in an implicit bouba-kiki experimental paradigm
Source: PLoS One. 2018 Dec 21;13(12):e0208874. doi: 10.1371/journal.pone.0208874 (PMC6303039; doi:10.1371/journal.pone.0208874)
Supplement: S1 Table — (DOCX) [file pone.0208874.s001.docx]

# S1 Table. List of pseudowords

| **Voiced plosives** | **Voiceless plosives** | **Sonorants 1** | **Sonorants 2** |
| --- | --- | --- | --- |
| abude | acape | amane | aloul |
| adibe | apipe | anoul | aloum |
| adude | apute | immal | amil |
| agade | atupe | immim | aname |
| badie | catte | imoul | iloum |
| baga | couk | innim | imale |
| bigu | cuke | lalla | imane |
| boube | icute | lami | linni |
| bougu | ikak | lanou | loume |
| bube | ikite | linou | louni |
| buda | ikuk | loula | lula |
| bugue | ipipe | loune | lulle |
| dagou | itape | lul | lumue |
| dide | kipou | lummu | malla |
| digou | pouke | lumou | mimue |
| doudi | pouki | malue | mounu |
| douga | puc | minnu | mune |
| dubu | pukou | muma | nal |
| gabe | pupue | munou | namie |
| gagou | puti | nalle | namue |
| gouga | puttu | nalli | nanu |
| gubi | quipe | namme | nilou |
| gugou | tapou | nannu | ninne |
| guibe | ticou | noune | noul |
| ibibe | touca | nune | numue |
| ibude | toucu | oulim | nunie |
| idabe | touki | oumul | oulil |
| idide | toutu | ounul | oumal |
| igabe | tutou | umam | oumum |
| ubibe | upipe | umine | uline |
| ugade | utape | unnim | umane |
| ugude | utate | unum | umoum |
