# Supplementary material for: Assessing sound symbolism: Investigating phonetic forms, visual shapes and letter fonts in an implicit bouba-kiki experimental paradigm
Source: PLoS One. 2018 Dec 21;13(12):e0208874. doi: 10.1371/journal.pone.0208874 (PMC6303039; doi:10.1371/journal.pone.0208874)
Supplement: S2 Table — (DOCX) [file pone.0208874.s002.docx]

# S2 Table. Properties of the lists of pseudowords

|  | **Voiced plosives** | **Voiceless plosives** | **Sonorants 1** | **Sonorants 2** |
| --- | --- | --- | --- | --- |
| **Structure (count)** | | | | |
| CVC | 6 | 6 | 6 | 6 |
| CVCV | 14 | 13 | 13 | 13 |
| VCVC | 12 | 13 | 13 | 13 |
| **Nb of letters** | | | | |
| Mean | 4,75 | 4,78 | 4,78 | 4,78 |
| Min | 4 | 3 | 3 | 3 |
| Max | 5 | 5 | 5 | 5 |
| **Nb of phonemes** | | | | |
| Mean | 3,81 | 3,81 | 3,81 | 3,81 |
| Min | 3 | 3 | 3 | 3 |
| Max | 4 | 4 | 4 | 4 |
| **Nb of orthographic neighbors** | | | | |
| Mean | 3,38 | 3,22 | 3,28 | 3,28 |
| Min | 0 | 0 | 0 | 0 |
| Max | 15 | 18 | 12 | 18 |
| **Nb of phonological neighbors** | | | | |
| Mean | 7,84 | 8,59 | 8,19 | 8,16 |
| Min | 0 | 0 | 0 | 0 |
| Max | 25 | 56 | 24 | 31 |
| **Average frequency of phonological neighbors** | | | | |
| Mean | 5,35 | 4,95 | 5,21 | 5,23 |
| Min | 0,00 | 0,00 | 0,01 | 0,00 |
| Max | 57,16 | 42,64 | 26,83 | 40,26 |
| **Average frequency of orthographic neighbors** | | | | |
| Mean | 2,66 | 3,33 | 3,54 | 3,26 |
| Min | 0,00 | 0,00 | 0,00 | 0,00 |
| Max | 40,68 | 43,43 | 50,60 | 41,28 |
| **Maximum frequency of phonological neighbors** | | | | |
| Mean | 27,80 | 70,86 | 74,15 | 56,17 |
| Min | 0,00 | 0,00 | 0,01 | 0,00 |
| Max | 289,63 | 573,72 | 453,95 | 537,44 |
| **Maximum frequency of orthographic neighbors** | | | | |
| Mean | 16,71 | 16,53 | 18,83 | 11,42 |
| Min | 0,00 | 0,00 | 0,00 | 0,00 |
| Max | 341,35 | 349,32 | 349,46 | 143,45 |
