# Supplementary material for: Assessing sound symbolism: Investigating phonetic forms, visual shapes and letter fonts in an implicit bouba-kiki experimental paradigm
Source: PLoS One. 2018 Dec 21;13(12):e0208874. doi: 10.1371/journal.pone.0208874 (PMC6303039; doi:10.1371/journal.pone.0208874)
Supplement: S3 Table — (DOCX) [file pone.0208874.s003.docx]

# S3 Table. List of words

| **Plosives** | **Sonorants** | **Mixed 1** | **Mixed 2** |
| --- | --- | --- | --- |
| agape | anime | amibe | aneth |
| agate | laine | anode | atoll |
| audit | lama | atome | atone |
| bader | lame | autel | banni |
| bague | lime | balai | bilan |
| battu | limer | bonne | bile |
| bec | lino | canot | caler |
| bidet | lune | coma | connu |
| biper | mamie | demi | culot |
| cabot | manie | dune | donne |
| cadet | manne | gaine | galop |
| coque | mener | gamma | gomma |
| coter | menu | goule | gone |
| coupe | mille | idole | goulu |
| daube | mime | item | hamac |
| digue | mimer | kilo | idem |
| dodo | minet | laque | imite |
| dodu | mini | ligue | lac |
| duc | minot | lubie | laide |
| duper | minou | lutte | lobe |
| gober | molle | mater | loti |
| godet | moule | matou | meute |
| gouda | moulu | midi | noter |
| otite | mule | nappe | nuque |
| papi | mulot | nette | patte |
| petit | muni | obole | peine |
| picot | naine | opine | peler |
| pub | nomme | paume | poney |
| tabou | nonne | piler | poule |
| tague | nul | polo | puma |
| tipi | nulle | puni | tamis |
| tique | ulule | tenu | utile |
