# Supplementary material for: Assessing sound symbolism: Investigating phonetic forms, visual shapes and letter fonts in an implicit bouba-kiki experimental paradigm
Source: PLoS One. 2018 Dec 21;13(12):e0208874. doi: 10.1371/journal.pone.0208874 (PMC6303039; doi:10.1371/journal.pone.0208874)
Supplement: S4 Table — (DOCX) [file pone.0208874.s004.docx]

# S4 Table. Properties of the lists of words

|  | **Plosives** | **Sonorants** | **Mixed 1** | **Mixed 2** |
| --- | --- | --- | --- | --- |
| **Structure (count)** | | | | |
| CVC | 10 | 15 | 10 | 11 |
| CVCV | 18 | 15 | 14 | 14 |
| VCVC | 4 | 2 | 8 | 7 |
| **Nb of letters** | | | | |
| Mean | 4,69 | 4,63 | 4,72 | 4,75 |
| Min | 3 | 3 | 4 | 3 |
| Max | 5 | 5 | 5 | 5 |
| **Nb of phonemes** | | | | |
| Mean | 3,69 | 3,53 | 3,69 | 3,66 |
| Min | 3 | 3 | 3 | 3 |
| Max | 4 | 4 | 4 | 4 |
| **Nb of orthographic neighbors** | | | | |
| Mean | 8,34 | 12,25 | 8,50 | 9,13 |
| Min | 1 | 3 | 0 | 0 |
| Max | 19 | 25 | 20 | 23 |
| **Nb of phonological neighbors** | | | | |
| Mean | 16,28 | 19,34 | 17,19 | 17,91 |
| Min | 1 | 5 | 2 | 3 |
| Max | 37 | 34 | 35 | 55 |
| **Average frequency of phonological neighbors** | | | | |
| Mean | 13,02 | 26,24 | 20,35 | 22,45 |
| Min | 0,08 | 0,05 | 0,01 | 0,08 |
| Max | 99,33 | 180,04 | 196,66 | 143,46 |
| **Average frequency of orthographic neighbors** | | | | |
| Mean | 21,16 | 33,69 | 25,50 | 35,94 |
| Min | 0,22 | 0,12 | 0,00 | 0,00 |
| Max | 347,75 | 453,51 | 475,49 | 968,89 |
| **Maximum frequency of phonological neighbors** | | | | |
| Mean | 413,48 | 1461,11 | 902,11 | 2008,79 |
| Min | 0,47 | 0,41 | 0,02 | 0,24 |
| Max | 4394,70 | 14946,48 | 14946,48 | 18188,15 |
| **Maximum frequency of orthographic neighbors** | | | | |
| Mean | 290,64 | 531,83 | 398,44 | 782,32 |
| Min | 0,34 | 0,14 | 0,00 | 0,00 |
| Max | 6882,16 | 9587,97 | 9587,97 | 23633,92 |
| **Word frequency in books** | | | | |
| Mean | 25,11 | 12,72 | 19,00 | 21,76 |
| Min | 0,00 | 0,07 | 0,00 | 0,14 |
| Max | 653,78 | 142,09 | 294,53 | 388,24 |
