# Supplementary material for: Assessing sound symbolism: Investigating phonetic forms, visual shapes and letter fonts in an implicit bouba-kiki experimental paradigm
Source: PLoS One. 2018 Dec 21;13(12):e0208874. doi: 10.1371/journal.pone.0208874 (PMC6303039; doi:10.1371/journal.pone.0208874)
Supplement: S1 Structure — (DOCX) [file pone.0208874.s007.docx]

# S1 Structure. Structure of the two datasets

**Dataset for pseudowords:**

- **Subject**: Subject ID (integer)
- **Stimulus**: Written form displayed on screen (string)
- **PhonologicalForm**: Phonological form corresponding to the written form, according to the convention used in *Lexique 3.81* (string)
- **Frame**: Frame ID, e.g. ‘Curve4’ or ‘Spike17’ (string)
- **FrameType**: Type of Frame, either ‘Curvy’ or ‘Spiky’ (string)
- **PloOrSon**: Whether the consonants are both ‘plosive’ or both ‘sonorant’ (string)
- **Voicing**: Whether the consonants are both ‘voiced’ or both ‘voiceless’ (string)
- **ConsonantCat**: Category of consonants, ‘sonorant’, ‘voiced_plosive’ or ‘voiceless_plosive’ (string)
- **TrialPosition**: position of the trial during the subject’s test (integer, from 1 to 256)
- **Font**: Font used for the written form, either ‘Gabriola’ or ‘Agency FB’ (string)
- **ResponseTime**: Response time in ms (double)
- **SOA**: Stimulus-onset asynchrony in ms (integer)
- **PrecedingResponseTime**: preceding response time in ms (double)
- **Structure**: Structure of the pseudoword, ‘CVC’, ‘CVCV’ or ‘VCVC’ (string)
- **Consonants**: Consonants used to build the pseudoword (string)
- **Vowels**: Vowels used to build the pseudoword (string)
- **NbLetters**: Number of letters in the pseudoword (integer)
- **NbPhonemes**: Number of phonemes in the pseudoword (integer)
- **StimuliList**: List of stimuli to which the pseudoword belongs (integer)
- **NbPhon**: Number of phonological neighbors (integer)
- **NbOrtho**: Number of orthographic neighbors (integer)
- **AvFrPhon**: Average frequency of occurrence of the phonological neighbors (double)
- **AvFrOrtho**: Average frequency of occurrence of the orthographic neighbors (double)
- **MaxFrPhon**: Maximum frequency of occurrence of the phonological neighbors (double)
- **MaxFrOrtho**: Maximum frequency of occurrence of the orthographic neighbors (double)
- **MedFrPhon**: Median frequency of occurrence of the phonological neighbors (double)
- **MedFrOrtho**: Median frequency of occurrence of the orthographic neighbors (double)
- **Gender**: Subject’s gender, either ‘M’ or ‘F’ (string)
- **Laterality**: Subject’s handedness, either ‘L’ or ‘R’ (string)
- **LateralityScore**: Subject’s laterality score, from -100 (left-handed) to 100 (right-handed) (double)

**Dataset for words:**

- **Subject**: Subject ID (integer)
- **Stimulus**: Written form displayed on screen (string)
- **PhonologicalForm**: Phonological form corresponding to the written form, according to the convention used in *Lexique 3.81* (string)
- **Frame**: Frame ID, e.g. ‘Curve4’ or ‘Spike17’ (string)
- **FrameType**: Type of Frame, either ‘Curvy’ or ‘Spiky’ (string)
- **PloOrSon**: Whether the consonants are both ‘plosive’, both ‘sonorant’ or ‘mixed’ (string)
- **Voicing**: Whether the consonants are both ‘voiced’, both ‘voiceless’ or ‘mixed’ (string)
- **ConsonantCat**: Category of consonants, ‘sonorant’, ‘voiced_plosive’, ‘voiceless_plosive’ or ‘mixed’ (string)
- **TrialPosition**: position of the trial during the subject’s test (integer, from 1 to 256)
- **Font**: Font used for the written form, either ‘Gabriola’ or ‘Agency FB’ (string)
- **ResponseTime**: Response time in ms (double)
- **SOA**: Stimulus-onset asynchrony in ms (integer)
- **PrecedingResponseTime**: preceding response time in ms (double)
- **Structure**: Structure of the word, ‘CVC’, ‘CVCV’ or ‘VCVC’ (string)
- **Consonants**: Consonants used to build the word (string)
- **Vowels**: Vowels used to build the word (string)
- **Voicing2ndConsonant**: Voicing of the first consonant, either ‘voiced’ or ‘voiceless’ (string)
- **Voicing2ndConsonant**: Voicing of the second consonant, either ‘voiced’ or ‘voiceless’ (string)
- **NbLetters**: Number of letters in the word (integer)
- **NbPhonemes**: Number of phonemes in the word (integer)
- **FrMovies**: Frequency of occurrences in movies (double)
- **FrBooks**: Frequency of occurrences in books (double)
- **StimuliList**: List of stimuli to which the word belongs (integer)
- **NbPhon**: Number of phonological neighbors (integer)
- **NbOrtho**: Number of orthographic neighbors (integer)
- **AvFrPhon**: Average frequency of occurrence of the phonological neighbors (double)
- **AvFrOrtho**: Average frequency of occurrence of the orthographic neighbors (double)
- **MaxFrPhon**: Maximum frequency of occurrence of the phonological neighbors (double)
- **MaxFrOrtho**: Maximum frequency of occurrence of the orthographic neighbors (double)
- **MedFrPhon**: Median frequency of occurrence of the phonological neighbors (double)
- **MedFrOrtho**: Median frequency of occurrence of the orthographic neighbors (double)
- **Gender**: Subject’s gender, either ‘M’ or ‘F’(string)
- **Laterality**: Subject’s handedness, either ‘L’ or ‘R’ (string)
- **LateralityScore**: Subject’s laterality score, from -100 (left-handed) to 100 (right-handed) (double)

In both datasets, **FrMovies, FrBooks**, **NbPhon**, **NbOrtho**, **AvFrPhon**, **AvFrOrtho**, **MaxFrPhon**, **MaxFrOrtho**, **MedFrPhon**, **MedFrOrtho**d are given in, or computed from, *Lexique 3.81*.
