# Supplementary material for: Assessing sound symbolism: Investigating phonetic forms, visual shapes and letter fonts in an implicit bouba-kiki experimental paradigm
Source: PLoS One. 2018 Dec 21;13(12):e0208874. doi: 10.1371/journal.pone.0208874 (PMC6303039; doi:10.1371/journal.pone.0208874)
Supplement: S1 Protocol — (DOCX) [file pone.0208874.s008.docx]

# S1 Protocol. Additional details of the experimental design

## Exclusion criteria for words

For words, we avoided all the sequences of graphemes with diacritics and implying the pronunciation of one of four French nasal vowels – [ɛ̃, œ̃, ɔ̃, ɑ̃]. For pseudo-words, the same constraints were applied but we additionally limited the selection of vowels to [a, i, y, u]. We also avoided the use of the written vowels “e” and “o” because of regional variations in their pronunciation[1]. The letter “e” was however used at the end of some strings when it was expected to be mute and helped produce “natural-looking” pseudo-words (e.g. “dide” – [did]). This also made pseudo-words slightly longer on average, although this was not a wanted outcome of the process.

## Keys to provide answers

Two keys were used by subjects to enter their choice whether a word or a pseudo-word was displayed: one on the left of the AZERTY keyboard (“q”), one on the right (“l”). In a ‘right-oriented’ version, “l” was used to answer “word” and “q” to answer “pseudo-word”. The ‘left-oriented’ version was the opposite. Participants were asked to choose which version they preferred.

## Constraints on the order of presentation of the stimuli

The order of presentation of the stimuli was constrained to avoid repetition effects as follows:

- no more than seven occurrences of one type of frame (i.e., spiky or rounded) in a row;
- no more than two occurrences of the same frame one after the other;
- no more than six words in a row, and no more than six pseudo-words in a row;
- no more than six exemplars of a given category of consonants in a row.

## References

1. Boula de Mareüil P, Woehrling C, Adda-Decker M. Contribution of automatic speech processing to the study of Northern/Southern French. Lang Sci. Elsevier Ltd; 2013;39: 75–82. doi:10.1016/j.langsci.2013.02.006
