# Supplementary material for: Assessing sound symbolism: Investigating phonetic forms, visual shapes and letter fonts in an implicit bouba-kiki experimental paradigm
Source: PLoS One. 2018 Dec 21;13(12):e0208874. doi: 10.1371/journal.pone.0208874 (PMC6303039; doi:10.1371/journal.pone.0208874)
Supplement: S1 Analysis — (DOCX) [file pone.0208874.s009.docx]

# S1 Analysis. Details of the statistical analysis

## Trimming of the response times

Response times for both pseudowords and words did not follow a Gaussian distribution, were bounded to the left and skewed on the right in favor of longer response times (see Fig 1). It therefore did not make sense to drop response times distant by more than 2.5 or 3 times the standard deviation from the mean response time, as this would have mostly trimmed longer response times, and hardly any shorter ones. This would have erased potentially important information contained in the thick right tail of the distribution, and would have possibly hidden some relevant effects.


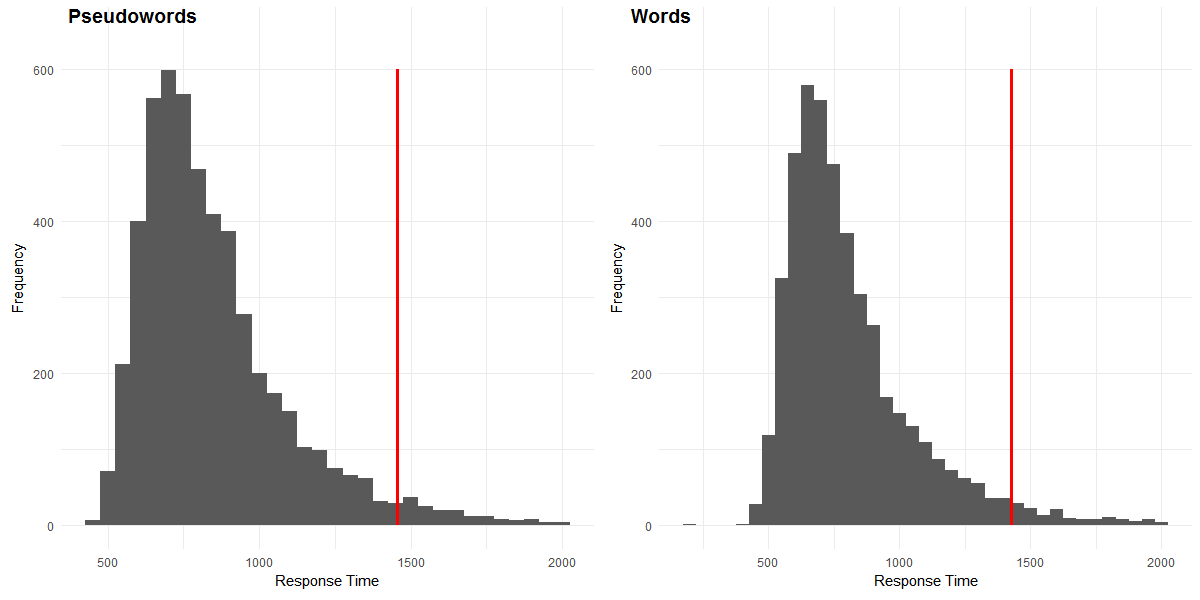


**Fig 1. Non-Gaussian distribution of response times for pseudowords and words.** Entries on the right of the red vertical line are distant by more than 2.5 times the standard deviation from the mean response time.

## Inclusion of additional predictors in the models

Baayen and Milin [1] have provided suggestions considering the appropriate modelling of response times. In particular, they have considered the trial position and the response time of the preceding trial as possible predictors, and ‘*found that including variables such as Trial and Preceeding RT in the model not only avoids violating the assumptions of linear modeling, but also helps improve the fit and clarifying the role of the predictors of interest’*. More precisely, including these effects help prevent temporal patterns of correlation of response times.

We therefore chose to consider these two predictors in our regression models, in addition to **Font**, **Type of frame**, **Category of consonants** and their interactions. For a subject’s first answer or after a failure to answer within 2000 ms, the preceding response time was replaced by their average response time during the experiment.

## Choosing an appropriate approach to model response times

### Inadequacy of a linear mixed effect model

The most straightforward regression modelling approach to response times is to consider a linear (mixed) model to relate them to predictors. However, an analysis of the residuals of such a model shows that the required assumptions of normality and homoscedasticity of the residuals are violated, as seen in Figure 2. The outputs of the model are therefore not to be trusted, even if it is robust to some extent to such problems.


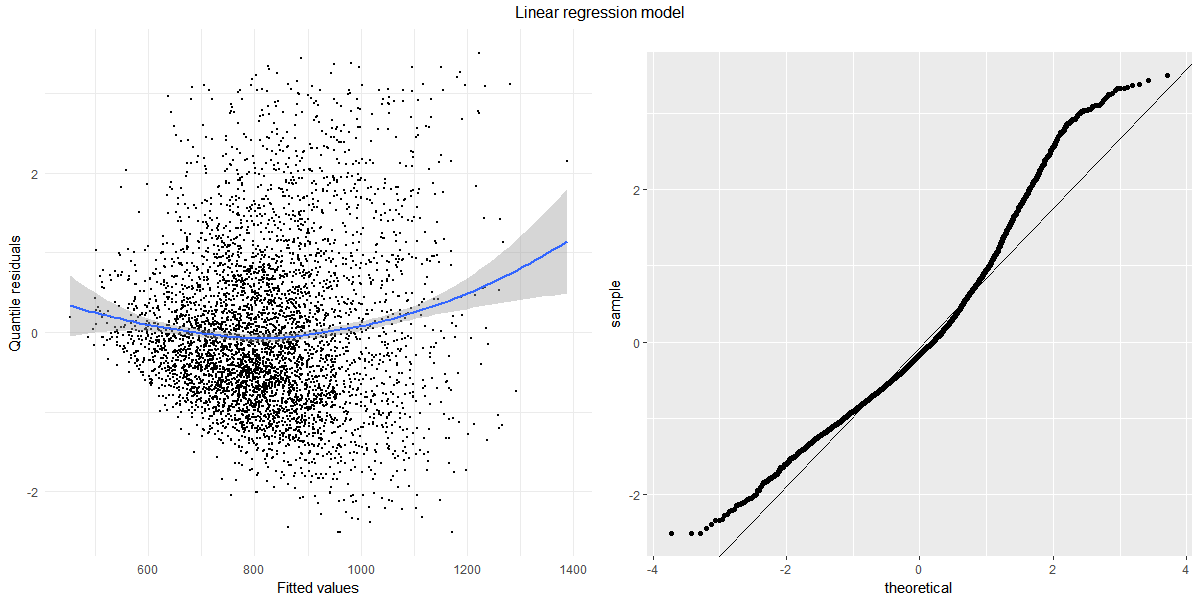


**Fig 2. Distribution of the residuals of a linear regression model for pseudowords against the linear predictor (left) and quantile-quantile plots of these residuals (right).** The left panel displays the heteroscedasticity, and the right panel the non-normality of these residuals.

A common solution to this issue is to apply a logarithmic or inverse transformation to the response times [2–5]. The resulting variable then often presents a Gaussian profile, which makes it fit for linear regression. However, as explained by Lo & Andrews [6], this approach is problematic, because ‘*statistically significant differences on the transformed metric are uninformative as to whether significant differences exist on the original untransformed metric and vice versa*’ (p. 3). In other terms, the significance of a predictor with respect to the logarithm or inverse of response times do not tell us about the significance of the relationship between this predictor and the untransformed response times.

All in all, linear regression models are therefore not well suited to response times.

### Shifting from linear to generalized linear mixed effect models

A possible solution to avoid transformation of the dependent variable consists in relying on generalized linear mixed regression models (GLMM), which offer more appropriate modelling of non-Gaussian distributions of the error terms, as well as a link function to relate the linear combination of predictors to the observed response. As suggested by Lo & Andrews, the *inverse Gaussian* and *Gamma* distributions make sense at a conceptual level for response times, as they adequately describe the time it takes for an event of interest to occur – pressing a key to answer in our case. Additionally, they advise choosing an *identity* link function – i.e., no transformation – to reflect the fact that models in mental chronometry directly link response times to mental processes.

We therefore first considered the ***glmer()*** function of the lme4 package, since it provided the *inverse Gaussian* (IG) and *Gamma* (GA) distributions to test with our data. We however experienced convergence issues, which given attempts with other datasets seemed to stem from the combination of these distributions with an *identity* link function. This led us to shift to generalized additive models for location, scale and shape (GAMLSS), as offered in the gamlss package, which did not suffer from such problems, and also allowed consider a much wider range of distributions for error terms.

### Generalized additive models for location, scale and shape

Generalized additive models for location, scale and shape (GAMLSS) [7–9] are an extension of generalized additive mixed models (GAMM) which allow to consider a wide range of options for the conditional distribution of the dependent variable (which corresponds to the distribution of error terms), while GLMM and GAMM are restricted to the exponential family of distributions [7]. Distributions offered in the gamlss.dist package differ on the number of parameters which can be modelled – up to four. These parameters are classically noted *µ*, *σ*, *ν* and *τ*, and correspond respectively to the location, the scale and the shape (the last two parameters) of the distribution. They are related, though not always equal, to the four moments of a distribution: mean, variance, skewness and kurtosis. They can be modelled, either with linear parametric, non-linear parametric or non-parametric (smooth) functions of the predictors.

As for the *Poisson* distribution for example, the only parameter that can be modelled is the location parameter, which is equal to the mean of the distribution. The scale and shape of the distribution cannot be modelled independently, since in a *Poisson* distribution the variance is equal to the mean, the skewness to the square root of the mean, and the excess kurtosis (the kurtosis minus 3) to the inverse of the mean. In the well-known Gaussian distribution, the mean and the variance of the distribution are independent from each other, and can be modelled separately, while the skewness and kurtosis are fixed.

We relied on GAMLSS to analyze the response times of our experiment and find an appropriate distribution for the location parameter, and left aside modelling options such as smooth terms. We modelled random effects with a specific smoothing function, in which a local maximum likelihood estimation is performed to shrink the fitted values of the factor predictor to the overall mean [8].

As previously, we first considered *IG* and *GA* distributions and followed the trimming procedure described in the methodological section. Although better than what was observed with a *Gaussian* distribution (NO), residuals were still not adequate enough to consider the adoption of either distribution, this for both pseudowords and words. It appeared that the problem had likely to do with the strong skewness of the distribution of response times. This led us to envisage other distributions, and especially the Generalized Gamma (GG) distribution, a 3-parameter distribution of which the *IG* and *GA* distributions are two specific instances, and the 4-parameter Johnson’s SU (JSU) distribution.

Table 1 and Table 2 summarize the adequacy of various distributions for pseudowords and words, respectively. Figure 3 and Figure 4 display the corresponding quantile-quantile plots of the residuals. For both pseudowords and words, the lowest AIC was obtained with the GA distribution. Normalized quantile residuals of this distribution, however, did not closely follow a normal distribution, as it was also the case for the IG distributions. The models with the GG and JSU distributions had higher AIC but a near-normal distribution of residuals. Among the two, the GG distribution led to a lower AIC, again both for pseudowords and words, and we therefore chose it as our target distribution, to be reported in the article. We, however, investigated the output of all models, and always found similar results for the **Type of Frame** × **Font** interaction depicted in the results of this study, although sometimes significance was not reached. This was a solid argument in favor of the existence of this interaction, beyond the singularity of a given model and a given dataset. Other interactions were significant in the JSU model, but did not match our hypotheses with respect to sound symbolism. The **Type of Frame** × **Category of Consonant** interaction found in the GG model for words was absent from the IG and GA models, and was unsupportive of sound symbolic hypotheses too.

**Table 1. Number of parameters, number of trimmed observations, global deviance, used degrees of freedom and AIC for GAMLSS models for pseudowords with various distributions (same predictors and predicted values).**

| *Distribution* | *Parameters* | *Deleted observations* | *Global deviance* | *df* | *AIC* |
| --- | --- | --- | --- | --- | --- |
| inverse Gaussian (IG) | 2 | 101 | 63,755 | 168.7 | 64,092 |
| Gamma (GA) | 2 | 124 | 63,551 | 168.7 | 63,889 |
| Generalized Gamma (GG) | 3 | 65 | 64,374 | 172.0 | 64,374 |
| Johnson’s SU (JSU) | 4 | 45 | 65,080 | 177.3 | 65,435 |


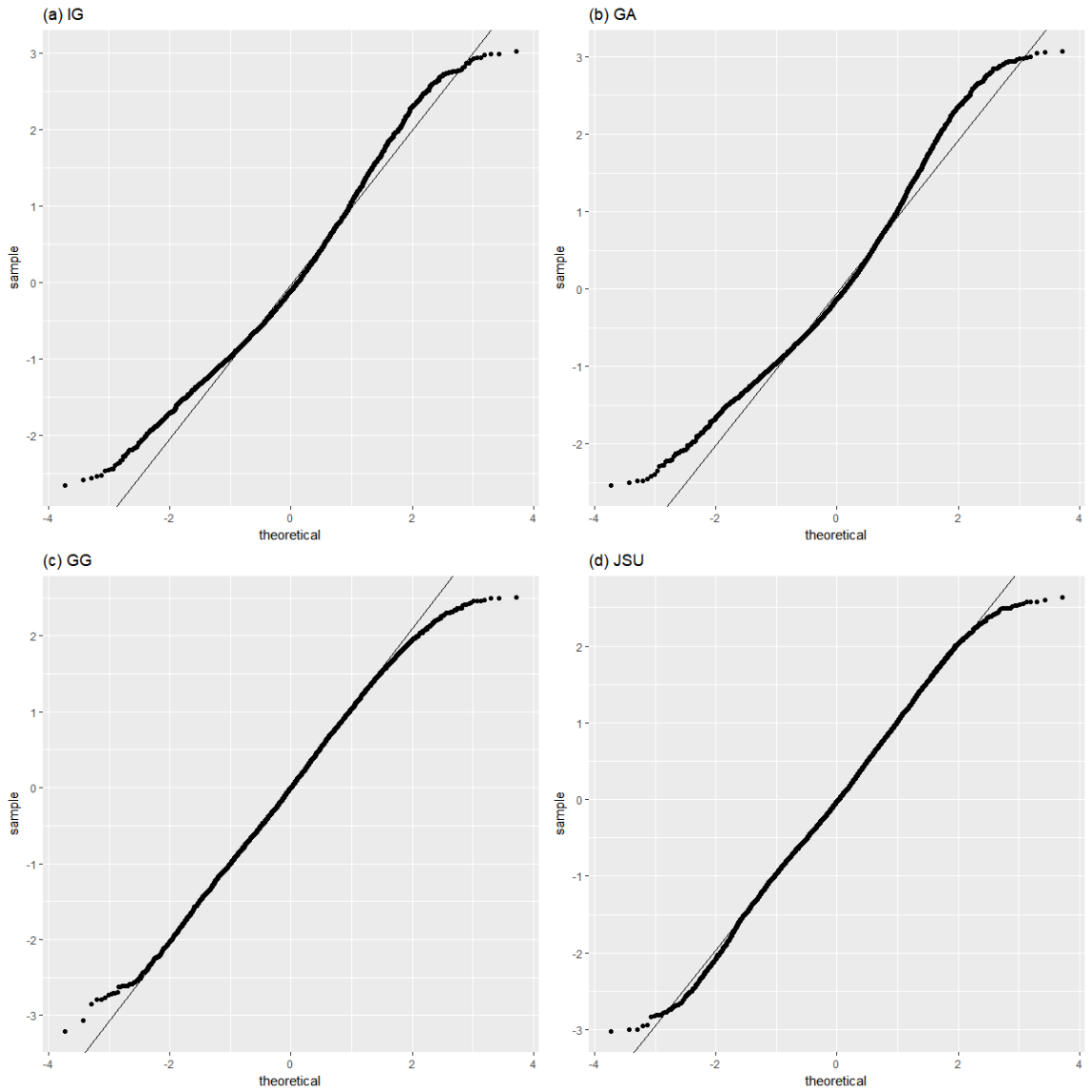


**Fig 3. Quantile-quantile plots of residuals for models for pseudowords with various distributions: IG (a), GA (b), GG (c) and JSU (d).**

**Table 2. Number of parameters, number of trimmed observations, global deviance, used degrees of freedom and AIC for GAMLSS models for words with various distributions (same predictors and predicted values).**

| *Distribution* | *Parameters* | *Deleted observations* | *Global deviance* | *df* | *AIC* |
| --- | --- | --- | --- | --- | --- |
| inverse Gaussian (IG) | 2 | 102 | 56,957 | 175.7 | 57,308 |
| Gamma (GA) | 2 | 124 | 56,772 | 176.2 | 57,125 |
| Generalized Gamma (GG) | 3 | 43 | 57,996 | 177.4 | 58,350 |
| Johnson’s SU (JSU) | 4 | 36 | 58,420 | 176.2 | 58,772 |


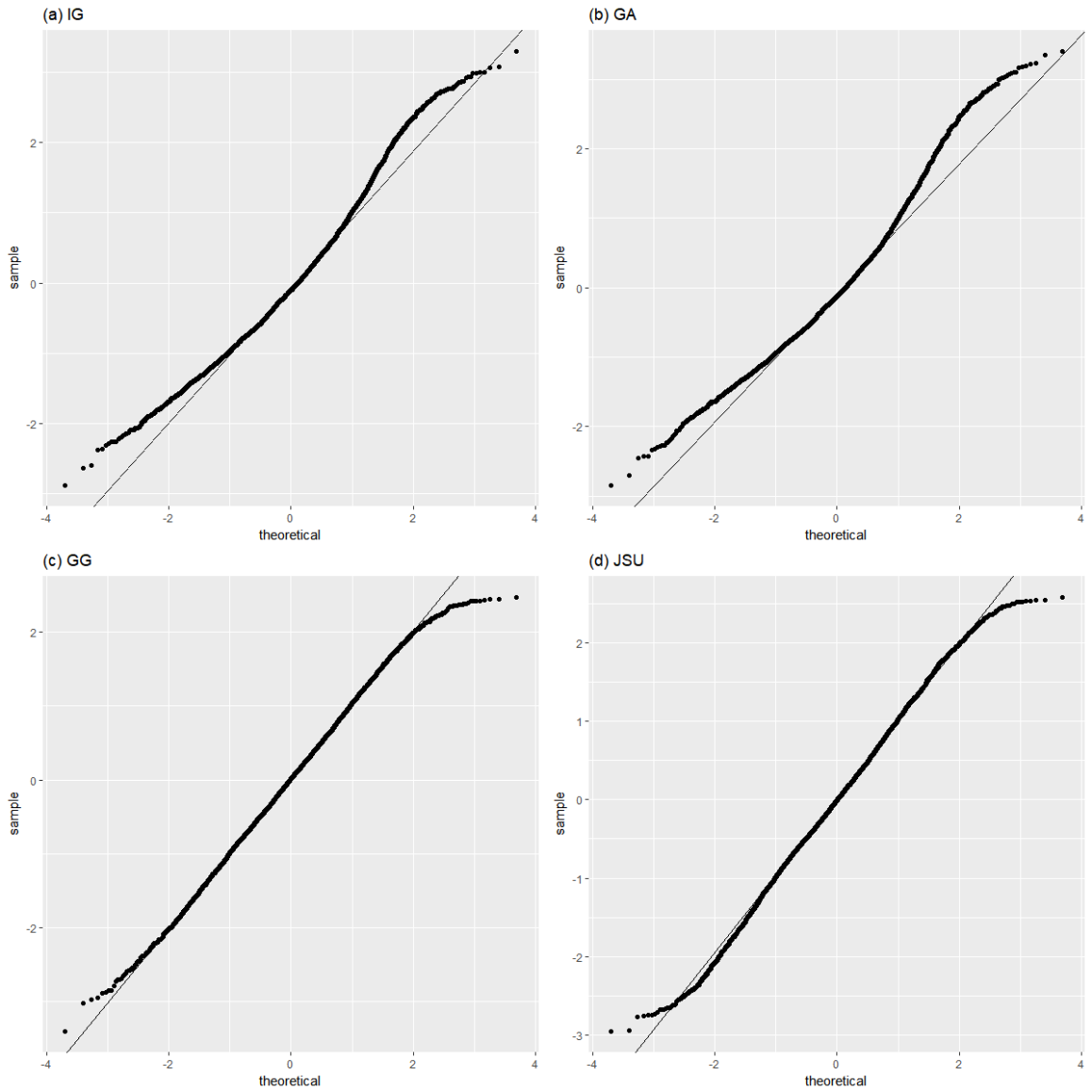


**Fig 3. Quantile-quantile plots of residuals for models for words with various distributions: IG (a), GA (b), GG (c) and JSU (d).**

### Checking the assumptions of the regression models

In addition to the normality of the residuals, other assumptions must be satisfied for a model to be valid: homoscedasticity of the residuals, linearity of the continuous fixed effects, absence of strong multicollinearity, and normal distribution of the modes of each random effect. We checked them for the GG GAMLSS models for pseudowords and words.

As an illustration, Figure 4 displays residuals of the model for pseudowords against the linear predictor to assess homoscedasticity. Figure 5 provides the quantile-quantile plots for the modes of the three random effects of the model for pseudowords. Finally, Figure 6 allows to assess the linear relationship between response times and both **Trial Position** and **Preceding Response Time**.

**
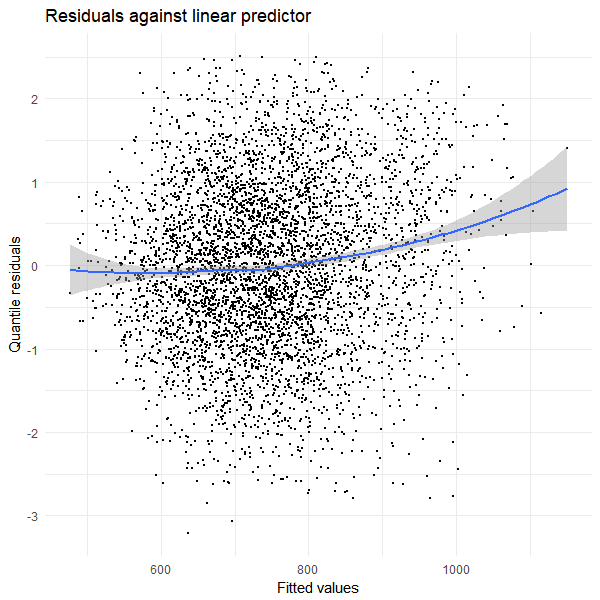
**

**Fig 4. Residuals of the GG GAMLSS model for pseudowords against the linear predictor.**


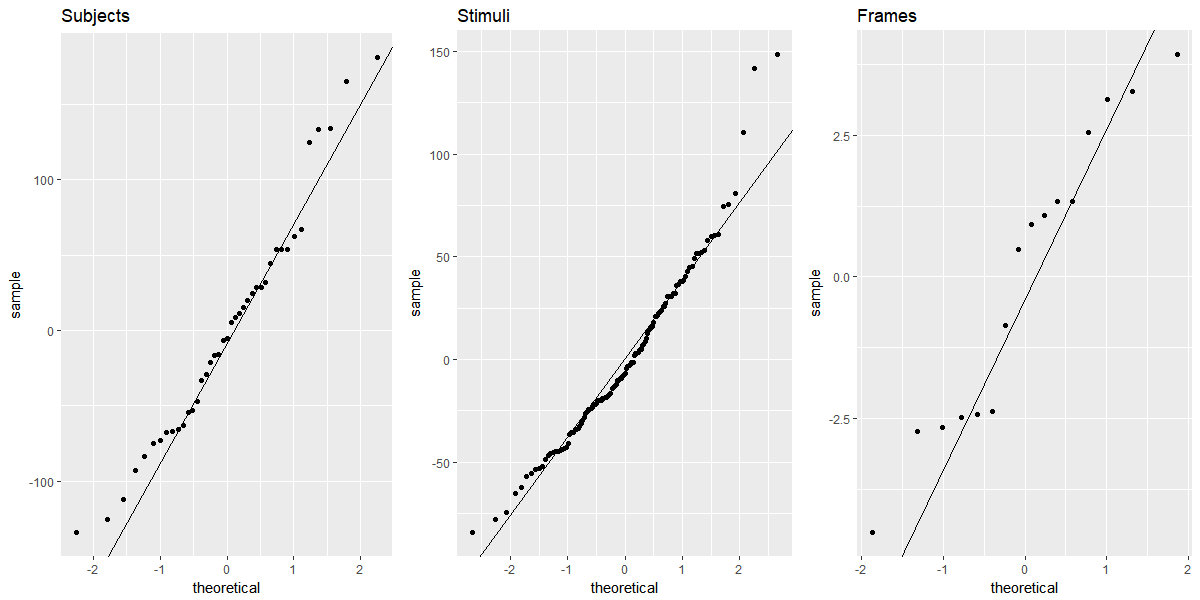


**Fig 5. Quantile-quantile plots for the modes of the three random effects of the GG GAMLSS model for pseudowords.** From left to right, the modes of the **Subject**, **Stimulus** and **Frame** random effects are displayed, respectively.

**
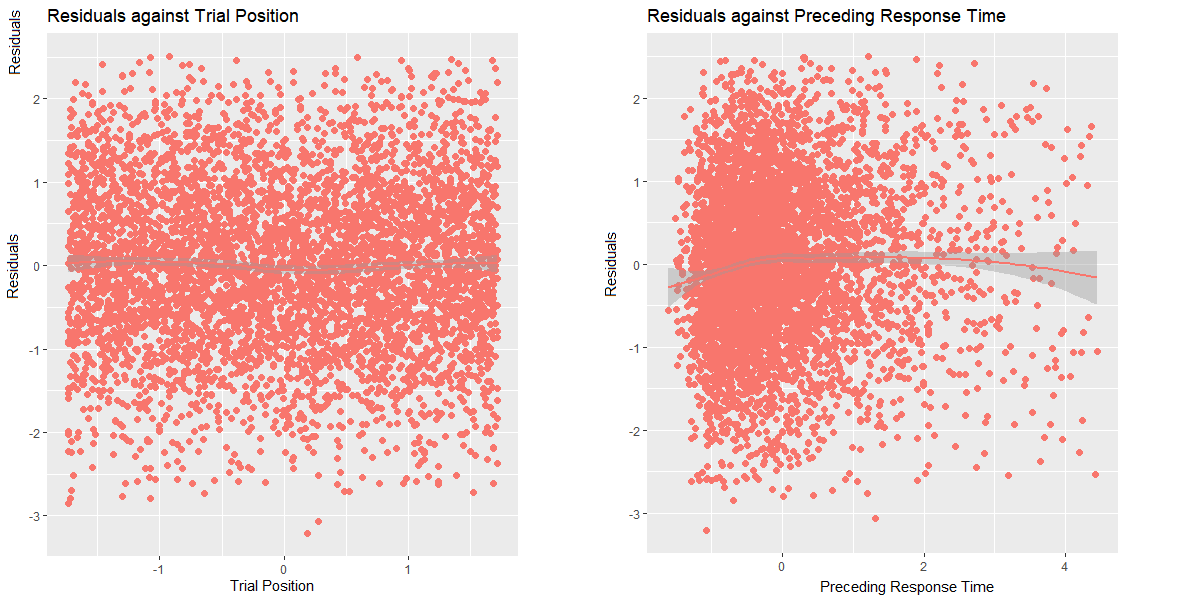
**

**Fig 6. Residuals of the GG GAMLSS model for pseudowords against Trial Position (left) and Preceding Response Time (right).**

## Assessing significance in GAMLSS models

As suggested by Stasinopoulos et al. [9], we relied on a series of Likelihood Ratio Test (LRT) to assess the significance of the predictors of the model, and in particular the significance of the three interactions of interest, namely **Type of Frame** **×** **Font**, **Type of Frame** **×** **Category of consonant**, **Font** **×** **Category of Consonant**. For each predictor, the deviance of the full model was compared to the deviance of the nested model without the selected predictor, testing the hypothesis that the two models have the same likelihood with the assumption that the difference of deviance is approximately χ² distributed. Dropping the target predictor without controlling for amount of shrinkage of the random effects would have led the nested model having a lower deviance than the full model, but random effects with higher degrees of freedom. In order to avoid this bias and produce correct differences between the two models in terms of degrees of freedom, we fixed the equivalent degrees of freedom of the random effects in the nested model to the values found in the full model. Doing so led to correct differences in degrees of freedom.

A predictor that appears to be significant must be interpreted cautiously if it is part of a significant higher-order interaction. Indeed, it is uneasy to interpret the effect of a variable when the size of this effect depends on the values of another variable – simple effects must replace main effects in this case. Because of this, we first assessed the significance of the triple interaction **Type of Frame × Font × Category of Consonant**. We had to verify that it was not significant to drop it and consider double interactions.

## References

1. Baayen RH, Milin P. Analyzing reaction times. Int J Psychol Res. 2010;3: 12–28. doi:10.1287/mksc.12.4.395

2. O’Hara RB, Kotze DJ. Do not log-transform count data. Methods Ecol Evol. 2010;1: 118–122. doi:10.1111/j.2041-210X.2010.00021.x

3. Warton DI, Lyons M, Stoklosa J, Ives AR. Three points to consider when choosing a LM or GLM test for count data. Methods Ecol Evol. 2016;7: 882–890. doi:10.1111/2041-210X.12552

4. Ives AR. For testing the significance of regression coefficients, go ahead and log-transform count data. Methods Ecol Evol. 2015;6: 828–835. doi:10.1111/2041-210X.12386

5. Zuur AF, Ieno EN, Elphick CS. A protocol for data exploration to avoid common statistical problems. Methods Ecol Evol. 2010;1: 3–14. doi:10.1111/j.2041-210X.2009.00001.x

6. Lo S, Andrews S. To transform or not to transform: Using generalized linear mixed models to analyse reaction time data. Front Psychol. 2015;6: 1–16. doi:10.3389/fpsyg.2015.01171

7. Rigby RA, Stasinopoulos DM. Generalized additive models for location, scale and shape (with discussion). Appl Stat. 2005;54: 507–554.

8. Rigby RA, Stasinopoulos DM, Lane PW. Generalized additive models for location, scale and shape. J R Stat Soc Ser C Appl Stat. 2007;23. doi:10.1111/j.1467-9876.2005.00510.x

9. Stasinopoulos M, Rigby RA, Heller GZ, Voudouris V, De Bastiani F. Flexible regression and smoothing using GAMLSS in R. CRC Press/Taylor & Francis Group; 2017.
